# Impact of Gene Modifiers on Cystic Fibrosis Phenotypic Profiles: A Systematic Review

**DOI:** 10.1155/2024/6165547

**Published:** 2024-10-16

**Authors:** Anastasia Ward, Ramil Mauleon, Chee Y. Ooi, Nedeljka Rosic

**Affiliations:** ^1^Faculty of Health, Southern Cross University, Coolangatta, Gold Coast, Queensland, Australia; ^2^Rice Breeding Innovations, International Rice Research Institute, Los Banos, Laguna, Philippines; ^3^School of Clinical Medicine, Discipline of Paediatrics & Child Health, Randwick Clinical Campus, UNSW Medicine & Health, UNSW, Sydney, Australia; ^4^Department of Gastroenterology, Sydney Children's Hospital, Randwick, New South Wales, Australia

**Keywords:** CFTR, cystic fibrosis, gene expression, genomic variations, modifier genes, phenotype, severity

## Abstract

Cystic fibrosis (CF) is a complex monogenic disorder with a large variability in disease severity. Growing evidence suggests that the variation observed depends not only on variations in the cystic fibrosis transmembrane conductance regulator (*CFTR*) gene but also on modifier genes. Utilizing five databases (including CINAHL, PubMed, Science Direct, Scopus, and Web of Science), a systematic review was conducted to examine the current literature on the known impacts of genomic variations in modifier genes on the CF disease progression, severity, and therapeutic response. A total of 70 full-text articles describing over 80 gene modifiers associated with CF were selected. The modifier genes included genes associated with the CFTR interactome, the inflammatory response, microbial profiles, and other genes affecting the critical physiological pathways of multiple organ systems, such as the respiratory and gastrointestinal systems. Limitations of the existing literature embrace the lack of clinical studies investigating pharmacogenetic impacts and the significance of gene modifiers on the CF clinical picture, including a limited number of replication and validation studies. Further investigations into other potential gene modifiers using genome-wide association studies are needed to critically explore new therapeutic targets and provide a better understanding of the CF disease phenotype under specific drug treatments.

## 1. Introduction

Cystic fibrosis (CF) is the most common debilitating and potentially lethal autosomal recessive genetic disorder, affecting an estimated 70,000–90,000 people globally [1]. CF is caused by mutations in the cystic fibrosis transmembrane conductance regulator (*CFTR*) genes, resulting in the CFTR protein becoming deficient, dysfunctional, or completely absent. The CFTR protein is responsible for transporting specific ions, such as chloride and bicarbonate, across the apical membranes and the modulation of other ion channels, such as the epithelial sodium channel (ENaC) [2]. In the disease state, a lack of or reduced transport of ions influences the transport of sodium, chloride, and water across the epithelial tissues, which leads to dehydrated, inspissated mucosal secretions. This, in turn, leads to impaired airway clearance and obstructive tubulopathy in other organs, which progresses to recurring cycles of inflammation and fibrosis [3]. As CFTR protein is expressed in “tubular” organs in the body, when its functionality is compromised like in CF, manifestations include bowel obstruction, exocrine and endocrine pancreatic insufficiency (PI), hepatobiliary disease, and male infertility [4].

Since the first discovery of the *CFTR* gene in 1989 [5], 2114 genetic variations have been reported and categorized into six classes, depending on their pathogenic role and impact on the CFTR protein stability and function (http://www.genet.sickkids.on.ca/cftr/, accessed on the 24th of September 2023). Pathophysiological profiles in CF patients may vary as *CFTR* gene variants may result in harmful (i.e., pathogenic), neutral (no effect), or even beneficial effects on CFTR protein function [6]. Whilst these different classes of *CFTR* mutations are associated with their own phenotypic variations, there is still extreme clinical heterogeneity within classes [7] and significant phenotypic variations in terms of the clinical picture and disease severity—even in the case of identical *CFTR* variants in both alleles. The most common disease-causing mutation is p.Phe508del, a class two variant occurring due to the deletion of three DNA bases within the *CFTR* encoding region, resulting in the removal of a single amino acid, phenylalanine (F). Over 60% of CF patients have the p.Phe508del variant present in both *CFTR* alleles, and ~90% of people living with CF have this variant in at least one allele [8]. Despite having the same genotype, as described in the case of the p.Phe508del homozygous variant, significant differences in pathophysiology profiles have been observed [9]. Monozygotic twins' studies confirmed the presence of additional genetic factors (i.e., gene modifiers [GMs]) influencing the disease phenotype [4, 10].

**Figure 1:**
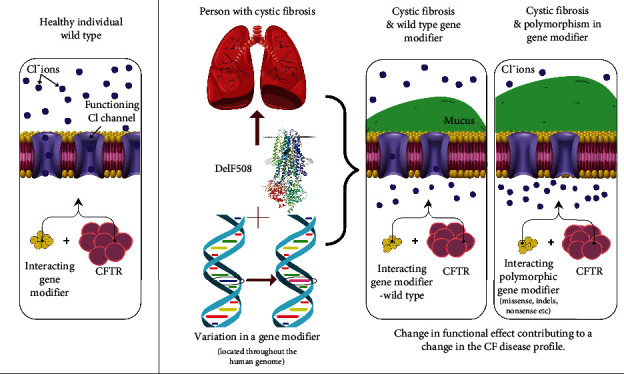
Schematic representation of an example gene modifier in conjunction with the p.Phe508del disease-causing variation.

Genetic variations in so-called GMs that are placed outside the primary disease-causing genes can also influence the disease phenotype [9]. GMs encode various molecules (e.g., transcription factors and transporters) that can enhance or suppress the severity of the disease outcomes without individually causing the actual disease [11]. The genetic polymorphisms of GMs may influence disease phenotype via interaction with the target gene directly (e.g., in CF, interacting with the *CFTR* gene), biochemically, or via functional interactions [9]. The degree to which the GM may affect the target gene can vary due to changes in penetrance, resulting in various disease phenotypes, even in the case of the same gene-causing variants. These non-*CFTR* GMs may impact multiple organs, including the intestine, liver, airways, pancreas, and others [4]. An additional negative impact on the respiratory system can be seen in CF (e.g., caused by the p.Phe508del mutation) when accompanied by GMs containing certain genetic variations (see schematic representation in Figure 1). For example, multiple studies have reported that functional variants in mannose-binding lectin (MBL), a protein of the innate immune system [12], have been found to influence lung disease severity, specifically declining lung function and earlier acquisition of *Pseudomonas aeruginosa* in CF patients [11]. Various GMs may also be present throughout the genome, influencing other systems and downstream processes and further impacting the CF clinical picture [11]. However, the significance of genomic variations of multiple GMs on the CF phenotype and its clinical consequences are still not well defined.

The image on the left (Figure 1) depicts a healthy individual with a variation in a GM. As visually described, no phenotypic effect is observed. To the right is the CF profile with the p.Phe508del mutation. The image describes the two possible scenarios: (i) the individual with a wild-type GM, showing no phenotypic effect and the normal accumulation of mucus in the CF profile and (ii) the individual living with CF with an interacting polymorphic GM. In conjunction with CFTR dysfunction, the mutated GM exacerbates mucus production, and as described in the final image, a greater buildup of mucus leads to a more severe disease. It is important to note that polymorphisms throughout the genome may affect the disease profile in CF, and this is not limited to one type or mutation in the *CFTR* gene.

### 1.1. Objectives

To date, no systematic review has been conducted to connect GMs with the entire CF phenotypes, in addition to the evidence on how GMs impact CFTR modulators (i.e., drugs used to treat defective CFTR protein) efficacy or absorption. This review is aimed at providing an updated overview of the effects of GMs relevant to CF on multiple systems, including pulmonary, gastrointestinal, endocrine, and cardiovascular systems.

### 1.2. Specific Objectives

i. Synthesize the current evidence of genetic variations in GMs relative to CF and determine the potential impacts of GMs on disease progression, pharmacokinetics, and phenotypic profiles associated with CF.
ii. Document the current research exploring variations in the *CFTR* gene, GMs, and gene modulator therapies.
iii. Identify potential GMs of interest for future drug therapies.

## 2. Methods and Materials

### 2.1. Information Sources and Eligibility Criteria

The SR was developed and conducted according to the Preferred Reporting Items for Systematic Reviews and Meta-Analyses (PRISMA) statement [13]. The SR was registered with PROSPERO, registration number CRD42022374657. Here, we detailed the methodology employed to conduct this SR according to the published protocol.

### 2.2. Search Strategy

The initial search strategy was drafted by listing keywords or phrases in the titles and abstracts and then the indexation of the records. The following search strategy terms were used and adapted for each database: (“cystic fibrosis” OR CFTR) AND (“single nucleotide polymorphism” OR “modifier gene^∗^” OR “candidate gene^∗^” OR “gene polymorphism” OR SNP OR mutation OR indel OR “genetic variation^∗^” OR “genomic variation^∗^”) AND (phenotype OR pathophysiolog^∗^ OR pathology OR aetiology OR etiology OR “clinical symptom^∗^” OR manifestation^∗^) AND (sever^∗^ OR progression OR decline OR accelerat^∗^).

Utilizing the initial string as a validation tool as to whether it identified the six chosen articles, the string was reviewed and refined in an iterative process until the standard string was established. A systematic search of five online databases using key phrases and Boolean operators was conducted to identify and analyze articles consisting of randomized controlled trials (RCTs), case-control studies, and observational studies. Five electronic bibliographic databases were included: CINAHL, PubMed, Science Direct, Scopus, and Web of Science. Searches were limited to articles in the English language or translated online. The timeframe was restricted to the last 13 years, from 2010 to 2023, to ensure the most up-to-date literature was retrieved. Details of the databases and search terms are recorded in Appendix A in the Supporting Information section. Reference lists from selected articles were also scanned and included if relevant.

### 2.3. Selection Criteria

The results obtained from the five electronic databases were compiled in EndNote. Any duplicates were then removed. The first reviewer (A.W.) appraised and evaluated the titles and abstracts for eligibility. Three authors (C.O., R.M., and N.R.) independently screened the full-text articles.

### 2.4. Data Extraction and Items

The first reviewer (A.W.) independently extracted the data using EndNote X9.3.3. A data extraction spreadsheet in Microsoft Excel (Version 2019) was designed and developed to chart and analyze the report and study data. Report data included the study characteristics such as authors, year published, title, and source of publication. The study data included the *CFTR* genotype, modifier gene, study method, country or population, age, sex, sample size, CFTR therapy or modulator, biological effects, disease outcome, and results. Study methods were categorized as qualitative versus quantitative and linkage versus association as defined by previously published classifications.

Once the data had been extracted, the initial string was then further utilized to locate previous studies conducted on the identified genes prior to 2010. Only studies evaluating the reported GMs from the 2010–2023 data were extracted from the second search to highlight historical replication or validation data on those genes.

### 2.5. Risk of Bias Assessment

The risk of bias and reporting quality of the included full-text studies was assessed using the Strengthening the Reporting of Observational Studies in Epidemiology (STROBE) instrument [14]. The instrument consists of a 22-item checklist, providing guidance on reporting observational and case-control studies whilst facilitating critical assessment and interpretation.

## 3. Results

### 3.1. Study Selection and Summary of Searches

**Figure 2:**
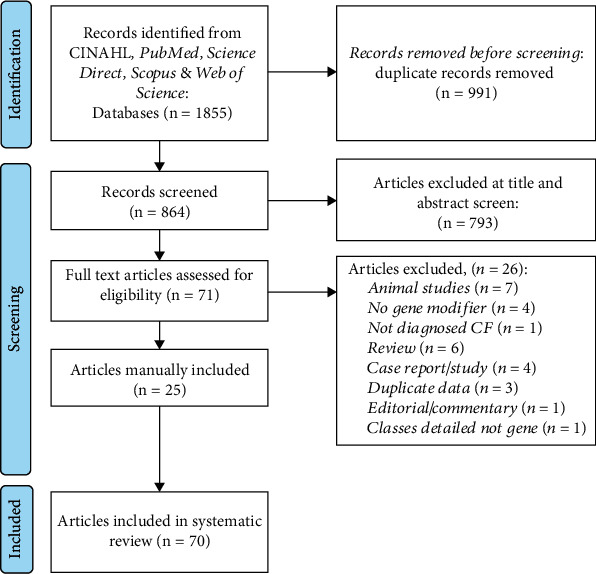
PRISMA flow diagram showing the screening and selection process of the included articles.

The systematic search identified a total of 1855 potentially relevant articles. A PRISMA flow diagram summarizing the results is depicted in Figure 2. After removing duplicates from the five databases, 991 studies were reviewed, assessing titles and abstracts against exclusion and inclusion criteria and research questions. In total, 793 articles failed to meet the inclusion criteria, thus being removed from the review. Seventy-two full-text remaining articles were screened and assessed for eligibility. Twenty-five articles were manually added. A total of 70 full-text articles were included in the review.

### 3.2. Description and Results of Individual Studies

The data extracted from the included 70 studies are comprehensively summarized in Table S1. Included were six genome-wide association studies (GWASs) [15–20], 27 candidate gene studies [7, 21–47], 16 case controls studies [48–63], seven cross-sectional studies [64–70], six observational studies [71–76], four whole exome sequencing (WES) retrospective studies [77–80], one transcriptome-wide association study [81], and two whole genome sequencing studies [82, 83].  Table 1 describes the list of modifier genes extracted from the included studies, including their clinical impact, polymorphism, and any interaction with therapeutics, if available.

#### 3.2.1. Studies' Characteristics

The sample size ranged from five in a WES study to 7840 in a whole genome sequencing study, with ages ranging from 2 to 77 years. The age range, as were the outcome measurements, varied across the studies and were inconsistent. Seven studies enrolled only adults as participants [31, 44, 56, 72, 73, 77, 82], four studies children exclusively [30, 65, 68, 69, 80], 50 had both children and adults [7, 15–18, 20, 22–26, 28, 29, 32, 33, 35, 36, 38–43, 46–55, 57, 58, 60, 62–64, 66–71, 74, 75, 78, 79, 81, 83, 84], and nine did not report the participant's age [19, 21, 27, 34, 37, 59, 61, 76].

As described in Tables 1, 2, and 3, the *CFTR* mutations consisted of 46 studies with a combination of classes represented and 26 studies that investigated polymorphisms in GMs exclusively in individuals living with the homozygous p.Phe508del mutation. Population data depicted 17 countries that had studies published. These included Brazil [26, 42, 64, 66–70], North America [20, 39, 51, 52, 55, 71, 77–79, 81, 82, 84], France [7, 21–23, 25, 31, 40, 56, 80], Italy [27, 49, 58, 59, 61, 63], Iran [53, 54, 57], Canada [30, 37, 41, 48, 72], Australia [43, 44, 65], Argentina [50], Belgium [33], Mexico [47, 60], Russia [62], Poland [28], and Switzerland [32, 46]. Eighteen studies used data from multiple countries [15–19, 24, 29, 34–36, 38, 45, 73–76, 83].

#### 3.2.2. GMs and Clinical Manifestations

The 70 identified studies described 81 modifier genes with 160 polymorphic sites. Of the 81 modifier genes described, 72 had only one study conducted. Seven GMs, including *ABCC1* [38, 55], *ADRB2* [47, 68, 69], *EDNRA* [29, 34], hemochromatosis (*HFE*) [43, 44], *HLA* [17, 83], interleukin 8 (*IL-8*) [65, 66], *TCF7L2* [15, 16], and *SLC11A1* [42, 70], had two studies conducted. The dynactin gene (*DCTN4*) [61, 78, 80] had three studies conducted. *MBL* [39, 47, 50, 60, 74] had five included studies. *SLC26A9* [16, 18, 19, 37, 42, 70, 84] had six studies. *SLC9A3* [17, 19, 30, 37, 42, 70, 83] had seven studies, and *SLC6A14* [7, 18, 19, 37, 40, 42, 70, 83] had eight studies conducted. The most commonly explored GM was tumor necrosis factor alpha (*TNF-α*), with nine studies conducted [24, 26, 47, 53, 54, 57, 60, 62, 65], affecting three systems: the respiratory, gastroenterological and musculoskeletal systems.

##### 3.2.2.1. Pulmonary Manifestations

**Figure 3:**
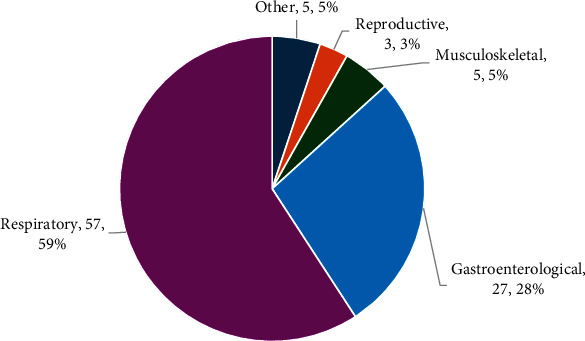
The frequency (count, %) of gene modifiers investigated associated with clinical manifestations in different systems in the included studies (*n* = 71).

A total of 45 GMs were reported to enhance the severity of the disease in contrast to 10 genes that were described as disease suppressors (Table 1). Four genes, depending on polymorphism, were both suppressors and enhancers. One gene described no effect. The outcomes focused mainly on lung disease, whereby 60 genes and 97 corresponding polymorphisms were reported to affect clinical outcomes in lung disease severity (Figure 3). Forty-five genes and 60 variations of the respiratory system were reported to enhance the disease severity. Nine genes and their corresponding polymorphisms, including *DNAH6* (rs1192269) [71], *GJA* (GJA4 variant) [73], *MBL* [39], macrophage migration inhibitor (*MIF*) (−794 CATT polymorphic repeats (MIF-CATT) [58], *PPP2R4* (rs3118625) [32], *PPP2R1A* (rs2162779) [32], *SLC6A14* (*rs12839137*) [7], *SCNN1B* (rs1391471777) [77], *TGBβ1* (rs1800469) [75], *TLR5* (rs5744168) [48], and *SCNN1D* (V541L and p579L) [77], were associated with less severe pulmonary disease.

A total of 19 GMs, consisting of 26 variants, of the respiratory system, including *ABCC1* (rs504348) ([38, 55]), *ADRB2* (Arg16Gly) [68, 69] (rs1042713) [47], *C3*(rs11569393) [41], *CAV2* (rs8940) [79], *CHI3L1* (rs4950928) [72], *DCTN4* (p.Tyr263Cys and rs35772018) [78, 80], *DEFB1* (52G>A, g-44C>G and g-20G>A) [61], *GCLC* (-129C>T and -3506A>G) [64], *GJA* (rs41266431) [73–75], *GSTT1* (GCLC-129C>T and GCLC-3506A>G) [64], *HFE* (C282Y and H63D) [43], IL-1*β* (−511 C/T) [65], *MBL* (rs11003125) [39, 47, 50], *MIF* (−794 CATT polymorphic repeats (MIF-CATT) [58], SLC6A14 (rs3788766) [37, 40, 42, 70], *SLC9A3* (rs17235416 and rs17563161) [37, 70], *T2R38* [49], *TMC6* (rs34712518) [79], and *TNF-α* (−308A>G) [24, 26], saw significantly earlier acquisition and increased airway colonization with *P. aeruginosa*. Where pulmonary disease severity was reported, this was measured by lung function tests such as FEV1. The detailed measurements of each study are described in Table S1.

##### 3.2.2.2. Gastroenterological Manifestations

Polymorphism in 25 genes, including *ADRB2* (Arg16Gly) [68, 69], *ATP12A* (rs61948108) [18], *HFE* (C282Y and H63D) [44], *CASR* [63], *CDKAL1* [16], *CDKN2A/B* [16], *CTRC* [63], *CYP11B2* [16], *IGF2BP2* (rs1470579 and rs4402960) [16], *KRT18P33* (rs11902125) [16], *LPHN3* (rs995447) [16], *MRSA* [35], *NCKAP1L* (rs475908) [16], *SERPINA1* (rs28929474 and rs17580) [22, 23], *SLC9A3* (rs17563161 and rs6864158) [19, 70], *SLC6A14* (rs3788766) [19, 37], SLC26A9 (rs7512462, rs7549173 and rs4077468) [18, 19, 37], *TCF7L2* (rs7903146 and rs34872471) [16], *TNF-α* (308GG and 3089GA [62] and 1031T/C [57], *TNF-β* (LT-*α*) (252AA and 252AG) [62], *GSTT1* (deletions) [64], *PRSS1* (rs3757377) [18, 63], *PRSS2* [63], *PTMA* (rs838440 and rs838455) [15] and *CHI3L1* (rs4950928) [72], were reported to have gastroenterological effects (Table 2 and Figure 3).

Specifically, *ADRB2* (Arg16Gly) [68, 69] was associated with PI. The mutations C282Y and H63D in the HFE protein were associated with increased rates of CF-related diabetes (CFRD), meconium ileus (MI), and distal intestinal obstruction syndrome (DIOS) [44]. *SLC9A3*'s polymorphism rs17563161 was associated with MI in two studies [37, 70] in addition to PI and diabetes mellitus, similar to deletions in the *GSTT1* gene [72]. The polymorphism, rs3788766, of the *SLC6A14* gene and rs7512462 of the *SLC26A9* gene was also associated with MI [37]. TNF-*α* and TNF-*β* (LT-*α*) and their associated polymorphisms, as described in Table 1, contributed to increased liver disease development, indicated by increased cirrhosis [57, 62]. Rs4950928, in the *CHI3L1* gene, was the only mutation associated with increased dysglycaemia [64].

##### 3.2.2.3. Reproductive, Musculoskeletal, and Other Manifestations

Two GMs, *EDNRA* and *TGF*, were associated with CBAVD penetrance, describing *EDNRA* (rs5225) as an enhancer in comparison to *TGF* (rs1982073 and rs1800471), where no effect was observed [34]. GMs of the Has-let-7e cluster (rs52196480) were reported to be associated with recurrent pregnancy loss [31]. Five GMs, including *GSTP1*, *TNF-α*, *TNF-β* (*LT-α*), *SLC6A14*, and *S1P*, were associated with the musculoskeletal system and an increased risk of osteoporosis and reduced bone density [56, 57, 62, 64, 70].

Only a single study [42] described the SNP-SNP interactions between the polymorphisms *SLC6A14* (rs3788766), *SLC26A9* (rs7512462), *SLC11A1* (rs17235416), and *SLC9A3* (rs17563161), depicting an earlier onset of gastroenterological symptoms. These interactions were reported to affect both the gastroenterological system, associated with PI, and the pulmonary system, associated with the presence of mucoid *P. aeruginosa*. Two other studies explored gene–gene interactions, specifically *GJA4* and *MBL* [74] and *GJA4* and *TGBβ1* [75]. No interactions were reported.

##### 3.2.2.4. GMs' Response to Therapies

A total of five studies evaluated the effects of GMs on response to therapies [7, 42, 67–69, 76]. Three studies reported the effects of GMs and bronchodilators [42, 67–69] in comparison to two studies that reported the effects of GMs on modulator therapy [7, 76]. Four pulmonary system genes, including *ADRB2* [68, 69], *IL-8* [67], *SLC26A9* [42, 76], and *SLC6A14* [42], were reported to influence the impact of therapies. The genes *ADRB2* and *IL-8* decreased the effects of bronchodilators, whereas *SLC6A14* positively influenced the efficacy of bronchodilators on the airways. Polymorphisms in *SLC26A9* [76] and SLC6A14 [7] were the only genes evaluated in therapeutic response to a *CFTR* modulator—ivacaftor/lumacaftor, resulting in an increased effect of this drug on the pulmonary system.

##### 3.2.2.5. Replication Studies of GMs Prior to 2010

**Figure 4:**
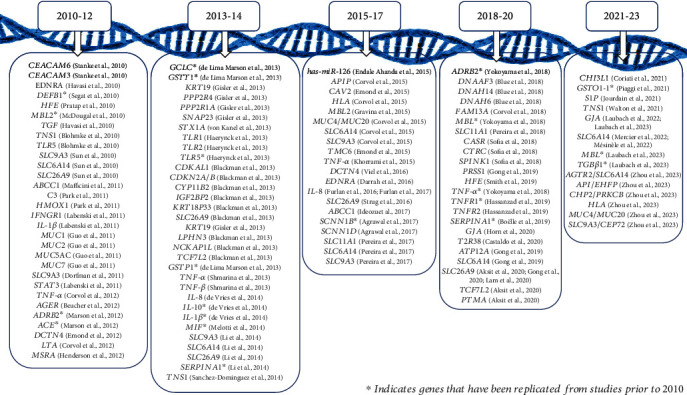
The timeline of the gene modifiers investigated in chronological order from 2010 to 2023.

Of the genes reported as GMs of CF from the date range investigated, 18 of these genes had previous studies conducted prior to 2010, as described in Table 4 and Figure 4. Over 58 investigations occurred between 1994 and 2010, whereby 27 studies replicated the current findings, and 18 studies were conflicting. Two systems, including the pulmonary and gastroenterological systems, were reported. Only two genes, *ACE* [112] and *SERPINA* [119], were investigated from the gastroenterological system, both evaluating the potential impacts of GMs on developing portal hypertension. The remaining 17 genes investigated GM pathobiological effects on pulmonary disease severity.

### 3.3. Risk of Bias

Table S1 provides the details on the level of risk and bias for the included studies. Issues including sample size numbers, varying outcome measurements, and lack of a comparison group all contribute to a moderate or high-risk bias for most studies. However, none were excluded because of the risk of bias.

## 4. Discussion

A growing body of evidence has been generated reporting the increasing associations between CF disease severity and genomic variations in non-*CFTR* genes. This SR reports the diverse array of over 80 GMs associated with CF, which is consistent with the numerous mechanisms involved in the pathogenesis of CF and variation in CF phenotypes.

### 4.1. Diversity of GMs Related to CF

The selected articles described numerous genes implicated in the pathogenesis of CF and genes hypothesized to interact with the CFTR protein or interactome profile. However, there is a distinct lack of up-to-date evidence surrounding GM polymorphisms that may influence the CFTR interactome profile. For example, studies have reported the different types of interactions between the CFTR protein and molecular chaperones (e.g., such as the *CANX* gene [120, 121] or *Aha1* [122].

### 4.2. Clinical Impacts of GMs

#### 4.2.1. GMs Directly Impacting the CFTR Protein and Ion Channel Activity

##### 4.2.1.1. Solute Carrier Family (SLC)

The SLC proteins, coexpressed with the CFTR protein in various epithelia, are multifunctional transmembrane proteins that mediate the transportation of monovalent and divalent anions [57]. Functional interaction and cross-talks between various SLC proteins with CFTR have been extensively studied and detail a reciprocal relationship based on the binding of the SLC sulfate transporter and antisigma factor antagonist (STAS) domain to the CFTR protein's regulatory domain [123]. The cytoplasmic STAS domain, a structural component of SLC proteins, is critical in intracellular trafficking, protein–protein interactions, and the regulation of chloride transport [124]. Genomic variants in the *SLC* genes have been reported to significantly affect or modulate the CFTR channel activity, subsequently impacting the CF phenotype in the major locations they are coexpressed, such as the inner ear, lungs, intestine, pancreas, and sperm cells [123]. Four genes of the *SLC* family, including *SLC11A1* [42, 70], SLC26A9 [16, 18, 19, 37, 42, 70, 84], *SLC6A14* [7, 18, 19, 37, 40, 42, 70], and *SLC9A3* [17, 19, 30, 37, 42, 70], have been identified as GMs of CF, influencing both positively and negatively MI, PI, CFRD, lung disease severity, the age of onset of *P. aeruginosa*, osteoporosis, lower age of onset of digestive symptoms, and nutritional status. The growing evidence reporting the regulation of CFTR by *SLC* genes in vitro suggests a potential novel therapeutic strategy in CF.

##### 4.2.1.2. ATP-Binding Cassette Subfamily C Member 1 (ABCC1)

*ABCC1* is a protein-coding gene that is structurally and functionally related to the *CFTR* gene, sharing its closest homology and both being members of the subfamily C member of transporter genes, the ATP-binding cassette genes [55]. *ABCC1* encodes for a multispecific organic anion transporter protein that plays a critical role in the efflux of several drugs and complements the *CFTR* gene [55]. Previous research evaluating epithelial cells in people living with CF (PLwCF) described a correlation between low *ABCC1* transcript levels and increased severity of CF [125]. The findings of Ideozu et al. [55] indicated that *ABCC1* expression may play a role in *CFTR* activity, but further research is required to fully understand the molecular underpinnings of how they interact. Previous research has also demonstrated the importance of genetic variations in the *ABCC1* gene, linking it to ovarian cancer, COPD, and major depression [126]. Further, Słomka et al. [126] also describe the frequency of *ABCC1* variants and the existence of ethnic differences. A critical factor when designing future studies evaluating *ABCC1* due to CF being a panethnic disease.

##### 4.2.1.3. Syntaxin 1A (STX1A)

STX1A, a member of the syntaxin superfamily, is described as a key molecule in ion channel regulation and intracellular trafficking [46]. Expressed in intestinal and respiratory cells, *STX1A* has been found to modulate CFTR functionality; thus, in CF, it further negatively regulates the remaining functionality of the dysfunctional protein [46]. von Kanel et al. [46] presented clinical and functional evidence that STX1A/C modified the CF disease phenotype and acts as a natural CFTR protein potentiator. Direct interactors with the CFTR protein, including SNAP23, KRT19, PPP2R1A, and PPP2R4 proteins, were also identified as lung disease modifiers in a longitudinal study [32].

##### 4.2.1.4. Genes of the ENaC

The ENaC is a Na^+^ ion regulator from the extracellular fluid into the cytoplasm and, in CF, is forced to become overactive, contributing to dehydration in the airways. ENaC is a heterotrimeric protein composed of *α* or *δ*, *β*, and *γ*-subunits. The *SCNN1A* and *SCNN1B* genes encode for the *α*- and *β*-subunit, whereas the *SCNN1D* gene encodes for the *δ*-subunit. In the WES study [77], the genes *SCNN1B* and *SCNN1D* were positively associated with lung function, and subsequently, survival, as they reduced channel activity, had a protective role in CF and provided a potentially new therapeutic strategy for disease management. In contrast, previous studies concluded that variants in the *SCNN1D* gene did not influence phenotypic severity in CF [127], and variants in the *SCNN1B* increased severity, particularly when exposed to tobacco smoking [106].

#### 4.2.2. GMs of Lung Function

Lung disease remains the primary source of mortality in CF [128]. Thus, genetic variations in GMs related to the respiratory system were the most prominently studied (Table 1) and replicated (Table 4). Respiratory manifestations in CF are caused by the inflammatory response in the respiratory tract due to chronic recurrent infection and mucus accumulation [129]. Several proinflammatory cytokines, such as TNF-*α*, IL-6, IL-8, and TGF*β*1, are consistently reported in the pathogenic role of CF [130]. Both TNF-*α* and TGF*β*1 have been studied and replicated multiple times prior to 2010. However, there was conflicting evidence surrounding the link between TGF*β*1 and pulmonary disease severity. In addition, cytokines IL-1*β*, IL-6, and TNF-*α* are linked to the initiation and persistence of pathologic pain [131]. In an aging CF population, pain is often an underestimated symptom and is encountered by 70% of adult patients [132, 133]. Research into the involvement of cytokines IL-1*β*, IL-6, and TNF-*α* and their variant's role in pain could potentially identify new therapeutic targets.

##### 4.2.2.1. TNF-*α*

TNF-*α*, found at high concentrations as part of the host defence mechanism and a direct biomarker of CF, is well documented in the literature and one of CF's most studied GMs [134]. TNF-*α* is associated with numerous phenotypes, including decreased pulmonary function, early infection of *P. aeruginosa*, decreased bone density, and an increase in liver disease development. Recent research has highlighted the effects of TNF-*α* on the efficacy of CFTR modulators [135]. However, to date, there have been no studies that explored the effects of variations in the *TNF-α* gene and the efficacy of modulator therapies. It is also reported as a GM in several other respiratory diseases, such as asthma and COPD [136, 137], and chronic inflammatory skin conditions such as psoriasis [138].

##### 4.2.2.2. Interleukin 8 (IL-8)

IL-8, a member of the CXC family of chemokines, promotes the inflammatory response in the airways [66]. In the studies included [65–67], three variants were investigated and associated with decreased pulmonary function. Comparable with other cytokines already described, IL-8 has been described in other GM studies of pulmonary phenotypic expression such as asthma [139], COPD [137], wheezing [140], and bronchiolitis [141], and also in other phenotypes such as lupus, breast cancer, ovarian, and prostate disease [66].

##### 4.2.2.3. MIF

MIF, a proinflammatory mediator, is significant in sustaining acute inflammatory responses by overriding the anti-inflammatory activity of glucocorticoids and inducing cytokine secretion such as TNF, IL-6, CXCL1, and CCL2 [142, 143]. MIF is associated with lung disease severity in CF, decreasing promoter activity, and is associated with a slower decline in lung function [58]. This is comparable to previous studies, where MIF was associated with mild pulmonary dysfunction and a low incidence of *P. aeruginosa* [105]. In autoimmune inflammatory diseases, such as multiple sclerosis, rheumatoid arthritis, systemic lupus, and asthma, *MIF* has also been reported as a modifier of interest, particularly in males [144].

##### 4.2.2.4. AGER Gene

Another proinflammatory protein of interest was the receptor for advanced glycation end products (RAGE). RAGE is expressed predominantly in respiratory epithelial cells, playing a major role in pulmonary homeostasis [21]. In CF, the expression of the RAGE protein is significantly increased in the airway neutrophils compared to the blood. In contrast, a lack of RAGE has been associated with pulmonary fibrosis [21]. A highly polymorphic gene, variants in *AGER*, which is responsible for encoding the RAGE protein, has been implicated in multiple diseases such as cancer, diabetes complications, COPD, acute respiratory distress syndrome, and cardiovascular disease [145]. With an aging CF population and diabetes being the most prevalent comorbidity, the *AGER* gene may be of clinical importance in the future.

#### 4.2.3. GMs of Infection Susceptibility

##### 4.2.3.1. Mannose-Binding Lectin 2 (MBL2) Gene

*MBL2* was one of CF's first genes to be investigated as a modulator of clinical severity [146]. A serum protein synthesized in the liver and functioning as a complement activator of the innate immune system, *MBL* has been extensively studied. Accumulation in the lungs during acute inflammation promotes phagocytosis, whereas in CF, which is typically characterized by chronic infection in the lungs by known pathogens such as *P. aeruginosa*, *MBL2* insufficiency can influence the early acquisition and chronic colonization of the microbes [50]. Five of the included studies investigated the effects of *MBL2* variants, focusing on the acquisition of infection and BMI [39, 47, 50, 60, 74]. In 2011, a review and meta-analysis conducted by Chalmers et al. [147] detailed the findings of over 16 studies, depicting, similar to our findings, inconsistent results and various methods of reporting. Chalmers et al. [147] also describe the potential for therapeutic supplementation, noting that randomized clinical trials have yet to be conducted to show any beneficial effect. Recent research has also investigated *MBL2* gene polymorphisms and their association with the severity of severe acute respiratory syndrome coronavirus (SARS-CoV-2) [148].

##### 4.2.3.2. HFE Gene

Two Australian studies investigated mutations in the *HFE* gene, which resulted in significantly lower lung function and increased rates of CFRD and DIOS [43, 44]. These are the only studies that investigated iron absorption related to the microbial profile and infection susceptibility in CF. Mutations in the *HFE* gene have also recently been associated with cluster headaches [149] and could be implicated in the prevalence of pain in CF.

##### 4.2.3.3. DCTN4

*DCTN4* was also investigated as a GM that may affect lung disease progression and survival [80]. The dynactin protein was associated with autophagy, and in CF, the defective DCTN4 protein was associated with age at the onset of chronic *P. aeruginosa* infection [80].

#### 4.2.4. GMs of the Gastroenterological System

##### 4.2.4.1. PI

The pancreas, one of the primary organs affected by the defective *CFTR* gene, is the most reliable phenotypic indicator of CFTR function [3]. Pancreatic manifestations were reported in four of the included studies [37, 42, 63, 68, 69], of which nine modifier genes were associated with pancreatic disease severity (*SLC*, *PRSS1*, *PRSS2*, *SPINK1*, *CTRC*, *CASR*, *CTSB*, *KRT8*, and *ADRβ2*). In recent in silico studies, *EPHX1*, *HLA*-*DQA1*, *HLA DQB1*, *DSP* and *SLC33A1*, *GPNMB*, *NCF2*, *RASGRP1*, *LGALS3* and *PTPN13* genes were also described as being potential GMs of the endocrine pancreas [150]. However, these genes are yet to be investigated to provide physiological relevance.

##### 4.2.4.2. MI

MI, a viscid obstruction of the terminal ileum developing in utero, is often the first indication of CF [151]. Six GMs, including *ATP12A*, *SLC26A9*, *SLC6A14*, *SLC9A3*, *MRSA*, and *PRSS1*, were reported to negatively influence MI in three of the included articles [18, 19, 35]. Limited evidence surrounding the roles of GMs in the development of MI and failure to replicate the studies that have been conducted are major limitations of these studies.

##### 4.2.4.3. CFRD

CFRD, recognized as Type 3c pancreatogenic diabetes, is the most prevalent comorbidity following pulmonary manifestations in PLwCF [152]. Associated with increased pulmonary exacerbations, a greater deterioration of lung function, and subsequent higher mortality rates, CFRD is a distinct clinical condition that requires a specific therapeutic approach [153]. CFRD outcomes were investigated in three of the included studies [15, 16, 43], of which variants (Table 1) of *TCF7L2*, *IGF2BP2*, *SLC26A9*, *CDKAL1*, *CDKN2A/B*, *CYP11B2*, *KRT18P33*, *NCKAP1L*, and *LPHN3* were all reported to be disease enhancers. The GM *TCF7L2* was of particular significance as it increased the risk of developing CFRD threefold and also significantly lowered the age of onset of CFRD [16]. SLC26A9, which interacts reciprocally or coexpresses with the CFTR protein [84], positively affected the age of onset of CFRD. Past studies have suggested that this GM variant may compensate for the dysfunctional CFTR protein in PLwCF and could be potentially used in the prevention or treatment of CFRD in CF [154].

##### 4.2.4.4. CF-Related Liver Disease

Cystic fibrosis liver disease (CFLD) is considered the third most common cause of CF death, affecting ~30% of CF patients [155]. The risk factors, prevalence, and evolution in CFLD are still unclear, with limited research conducted on the impact of GMs on liver disease [22, 23]. The GMs explored related to CFLD [22, 23, 62, 64] were also linked to pulmonary emphysema [156], liver disease, and COPD [157]. In a landmark study by Bartlett et al. [119], the *SERPINA1 Z* allele was also recognized as a significant GM and a risk factor for liver disease in PLwCF. However, no further studies on the *SERPINA* gene have been conducted in conjunction with in silico studies to explore any other polymorphisms in relation to CF further. In addition, earlier studies on the *ACE* gene were shown to increase the chances of developing portal hypertension [112], yet they have not been further investigated in recent years. Further research is required on hepatic manifestations and the potential role of modifier genes, specifically in pharmacogenetics, whereby precision medicine may reduce potential hepatotoxicity.

### 4.3. Future Therapies

Pharmacotherapy of CF includes a diverse range of therapeutics traditionally aimed at treating the complications of CF. This includes pharmacological groups such as anti-inflammatories (nonsteroidal anti-inflammatory drugs and glucocorticoids), mucoactive drugs (e.g., bronchodilators, mannitol, and dornase alpha), antibiotics, and CFTR modulators (CFTR potentiator [ivacaftor] and three CFTR correctors [elexacaftor, lumacaftor, and tezacaftor]) [158]. CFTR modulators act more upstream on the pathogenesis cascade and are a targeted treatment based on specific mutations [159]. Recent research has highlighted the varied response to modulator therapies, even with the same class mutation, whereby complex alleles and combinations of variants are currently being explored [160]. In an era of personalized medicine, conducting theratyping based on genotype is critical. Theratyping is the term used to describe or group *CFTR* variants depending on their functional impacts on the CFTR protein and the subsequent response to therapy, such as corrector and potentiator compounds [161]. According to the Cystic Fibrosis Foundation Patient Registry's Annual Data Report, 87.6% of the US CF population are currently prescribed modulator therapies [162]. This review highlights the need for increased research in the area of theratyping, whereby these studies have only been conducted in a single study and need to be replicated to provide validation. Further, there was no data on the efficacy of Trikafta, the combination of ivacaftor, elexacaftor, and tezacaftor, in CF patients with polymorphisms in the *CFTR* gene or modifier genes. There are numerous applications of theratyping in CF research, such as optimizing modulator selection and the selection of participants in clinical trials [161]. Due to the vast number of genes and subsequent protein–protein interactions impacting drug metabolism and transport, genetic variations and their significance cannot be underestimated. Multiple studies included in this SR [42, 67–69, 76] showed that genetic variations, particularly in metabolizing enzymes and transporter substrates, inhibitors, and inducers, can markedly affect the severity of potential drug interactions and must be considered when prescribed.

### 4.4. Limitations

There were some limitations in the evidence generated from this SR, as the majority of selected studies have primarily focused on individual polymorphisms or employed a single locus analysis strategy whereby the variants are tested individually in association studies with a phenotype. However, this approach is insufficient in explaining the disease manifestations or susceptibility in a complex disease like CF by failing to identify the interactions with additional factors, such as epigenetic and environmental factors [163]. Very few publications in CF GM research have incorporated interaction testing. One study [70] described the interactions between the polymorphisms in multiple genes, including *SLC6A14*, *SLC26A9*, *SLC11A1*, and *SLC9A3*, which resulted in an earlier onset of digestive symptoms. Due to still limited evidence, further research is required to evaluate multiple GM interactions when testing for associations at a given locus and linking it to the CF phenotype. Further, a major factor includes the limited data on clinical impact and, in conjunction, the variability of outcome measures when the clinical impact is reported. In particular, the most common outcome measure reported, the FEV1 or Tiffeneau–Pinelli index, was observed to have different reference equations and often did not report the frequency or timing of the measurement. Table 4 also indicated several conflicts comparing the outcomes of different studies. Further replication studies should be conducted to validate the findings in more extensive studies with increased statistical power.

## 5. Conclusion

Multiple studies reported significant associations between polymorphisms in modifier genes and the *CFTR* gene and their impact on the CF phenotype and disease severity. This review highlights the existing up-to-date evidence, including replication studies prior to 2010, about these interactions and their potential to be further explored towards improved clinical outcomes. The continued identification and investigation of GMs are critical for providing new therapeutic targets and the development of biomarkers to stratify populations. Consequently, patients with an increased risk of disease severity could be treated with more aggressive therapies and the application of early intervention strategies. Therefore, further studies are needed to replicate and validate the previous studies that were described in the SR and potentially identify and analyze new modifier genes. Future studies, including whole genome association studies with sufficient power and pharmacogenetic analyses with adequate study design, should provide better-quality data on the role of GMs in CF. In addition, testing the study populations for all known GMs of interest compared to a single gene could increase the reliability of positive associations and add to the new knowledge concerning GM interactions with different *CFTR* gene variants. Finally, these GM-based findings may be used to improve the treatment options and provide better-quality personalized care for the people living with CF.

## Figures and Tables

**Table 1:** Summary of investigated gene modifiers associated with the respiratory system and their characteristics, including relevant references.

**Name of gene**	**Gene abbreviation**	**Polymorphism (if described—Indicated as dbSNP rs ID, the base/amino position and nucleotide/amino acid change, or variant type)**	**Enhancer or suppressor GM**	***CFTR* genotype**	**Clinical impact**	**Influence on therapies (where available)**	**Studies conducted**	**References**
ATP binding cassette subfamily C member 1	*ABCC1*	rs504348 (5′FR/G-260C)	Enhancer	p.Phe508del homozygous	↑Earlier acquisition of *P. aeruginosa* infection Chronically colonized by *P. aeruginosa* ~6 years earlier		CGA	Mafficini et al. [38]
			Enhancer	p.Phe508del homo and heterozygous	↑Colonization by *P. aeruginosa* and mucoid *P. aeruginosa* in the pancreatic insufficient group		CC	Ideozu et al. [55]

Adrenoceptor beta 2	*ADRB2*	rs1042713	Enhancer	p.Phe508del homozygous	↑PD severity ↑Airway colonization—*P. aeruginosa*—lower age at the first isolation	↓Effects of bronchodilators	CGA	Yokoyama et al. [47]
							CSS	Marson et al. [68, 69]

Angiotensin I converting enzyme	*ACE*	rs4646994 (D/I polymorphism)	Enhancer	p.Phe508del homo and heterozygous	↑PD severity		CSS	Marson et al. [68, 69]
					↑Earlier acquisition and colonization of *Burkholderia cepacia*			
					↑Risk for chronic infection with *B. cepacia*			

Advanced glycosylation end-product-specific receptor	*AGER*	rs1800625 (−429T/C)	Enhancer	p.Phe508del homozygous	↑PD severity by increased RAGE expression		CGA	Beucher et al. [21]

Angiotensin II receptor type 2	*AGTR2/SLC6A14*	rs12009976	Enhancer	p.Phe508del homo and heterozygous	↑PD severity		WGS	Zhou et al. [83]

APAF1 interacting protein	*APIP*	rs10742326	Enhancer	p.Phe508del homo and heterozygous	↑PD severity		GWAS	Corvol et al. [17]
	*API/EHFP*	rs485845	Enhancer	p.Phe508del homo and heterozygous	↑PD severity		WGS	Zhou et al. [83]
		rs12793173	Enhancer	p.Phe508del homozygous	↑PD severity		WGS	Wright et al. [20]

Caveolin 2	*CAV2*	rs8940	Enhancer	p.Phe508del homo and heterozygous	↑Earlier acquisition of *P. aeruginosa* infection		WES	Emond et al. [79]

Complement factor 3	*C3*	rs11569393	Enhancer	p.Phe508del homo and heterozygous	↑PD severity ↑Airway colonization—With *P. aeruginosa*		CGA	Park et al. [41]

Chitinase 3 like 1	*CHI3L1*	rs4950928	Enhancer	p.Phe508del homo and heterozygous	↑PD severity measured by FEV1 score ↑Infection susceptibility—Increased colonization with *P. aeruginosa*		OB	Coriati et al. [72]

Calcineurin B homologous protein 2	*CHP2/PRKCB*	rs194788	Enhancer	p.Phe508del homo and heterozygous	↑PD severity		WGS	Zhou et al. [83]

Dynactin subunit 4	*DCTN4*	rs11954652	Enhancer	p.Phe508del homozygous	↑PD severity ↓Survival rate ↑Chronic *P. aeruginosa* infection		WES	Viel et al. [80]
		rs35772018	Enhancer	p.Phe508del homozygous	↑PD severity ↓Survival rate ↑Chronic *P. aeruginosa* infection			Viel et al. [80]
			Enhancer	p.Phe508del homo and heterozygous			WES	Emond et al. [78]

Defensin beta 1	*DEFB1*	g-52G>A, −44C>G g-20G>A	No effect	p.Phe508del homo and heterozygous			CC	Segat et al. [61]

Dynein axonemal assembly factor 3	*DNAAF3*	rs58824375	Enhancer	p.Phe508del homo and heterozygous	↑PD severity		OB	Blue et al. [71]
Dynein axonemal heavy chain 14	*DNAH14*	rs3856154 rs950210	Enhancer		↑PD severity		OB	
Dynein axonemal heavy chain 6	*DNAH6*	rs1192269	Suppressor		↓PD severity		OB	

Endothelin receptor type A	*EDNRA*	rs5335	Enhancer	F508del homozygous	↑PD severity		CGA	Darrah et al. [29]

Family with sequence similarity 13 member A	*FAM13A*	rs7682431	Enhancer	p.Phe508del homozygous	↑PD severity ↑COPD		CGA	Corvol et al. [25]

Glutamate-cysteine ligase catalytic subunit	*GCLC*	129C>T	Enhancer	p.Phe508del homo and heterozygous	↑Airway colonization—With mucoid *P. aeruginosa* ↑PD severity		CSS	de Lima Marson et al. [64]
		3506A>G	Enhancer		↑Frequency of nonmucoid *P. aeruginosa*			

Gap junction protein alpha 1	*GJA*	GJA4 variant	Suppressor	p.Phe508del homozygous	↓PD severity		OB	Horn et al. [73]
		rs41266431	Enhancer	p.Phe508del homozygous	↑PD severity associated with ↓survival to end-stage lung disease, presented with worse LFTs ↑*P. aeruginosa* colonization		OB	Horn et al. [73], Laubach et al. [74], and Laubach et al. [75]

Glutathione S-transferase omega 1	*GSTO1-1*	A140D	Enhancer	p.Phe508del homo and heterozygous	↑PD severity associated with lower levels of the anti-inflammatory mediators PGE2 and 15(S)-HETE and with lower values of the FEV1/FVC ratio ↑COPD		CC	Piaggi et al. [59]

Glutathione S-transferase theta 1	*GSTT1*	Deletions	Enhancer	p.Phe508del homo and heterozygous	↑PD severity		CC	de Lima Marson et al. [64]
					↑Risk of cancer			
		GCLC-129C>T	Enhancer	p.Phe508del homo and heterozygous	↑PD severity—Higher frequency of the *P. aeruginosa* mucoid		CC	de Lima Marson et al. [64]
		GCLC-3506A>G	Enhancer	p.Phe508del homo and heterozygous	↑PD severity—Higher frequency of the *P. aeruginosa*		CC	de Lima Marson et al. [64]

Heme oxygenase 1	*HMOX1*	rs2071749	Enhancer	p.Phe508del homo and heterozygous	↑PD severity		CGA	Park et al. [41]
		rs2071746	Enhancer	p.Phe508del homo and heterozygous	↑PD severity		CGA	Park et al. [41]

Human leukocyte antigen	*HLA*	rs116003090	Enhancer	p.Phe508del homo and heterozygous	↑PD severity		GWAS	Corvol et al. [17]
		rs9268860	Enhancer	p.Phe508del homo and heterozygous	↑PD severity		WGS	Zhou et al. [83]

MicroRNA 126	*has-miR-126*	rs376594280	Enhancer	p.Phe508del homozygous	↑PD severity ↑Inflammation		CGA	Endale Ahanda et al. [31]
		rs41275794			↑Inflammation		CGA	
		rs12976445			↑Inflammation		CGA	

Homeostatic iron regulator	*HFE*	rs1800562 (C282Y)	Enhancer	p.Phe508del homo and heterozygous	↑PD severity including lower FEV(1) % ↑FEV(1) decline		CGA	Smith et al. [44]
			Enhancer	p.Phe508del homo and heterozygous	↑PD severity ↑Airway colonization with *P. aeruginosa*		CGA	Pratap et al. [43]
		rs1799945 (H63D)	Enhancer	p.Phe508del homo and heterozygous	↑PD severity ↑Airway colonization with *P. aeruginosa*		CGA	Pratap et al. [43]

Interferon gamma receptor 1	*IFNGR1*	rs1327475 rs1800896 rs9376268 rs9376269	Enhancer	p.Phe508del homozygous	↑PD severity		CGA	Labenski et al. [36]

Interleukin 10	*IL-10*		Enhancer	p.Phe508del homo and heterozygous	↑Airway colonization ↑PD severity		CSS	de Vries et al. [65]
Interleukin 1 beta	*IL-1β*	−511 C/T	Enhancer		↑Airway colonization with mucoid *P. aeruginosa* ↑PD severity		CSS	
		rs3917356 rs4848306	Enhancer	p.Phe508del homozygous	↑PD severity		CGA	Labenski et al. [36]

Interleukin 8	*IL-8*	rs4073	Enhancer	p.Phe508del homozygous	↑Airway colonization ↑PD severity		CSS	de Vries et al. [65], Furlan et al. [66] and Furlan et al. [67]
					Potential ↑severity of lupus nephritis, periodontitis, breast cancer, prostate diseases, and ovarian diseases	↓Effects of bronchodilators		
		rs2227306	Enhancer	p.Phe508del homozygous	↑PD severity	↓Effects of bronchodilators	CSS	Furlan et al. [66] and Furlan et al. [67]
		rs2227307	Enhancer	p.Phe508del homozygous	↑PD severity	No effects on bronchodilators	CSS	Furlan et al. [66] and Furlan et al. [67]

Keratin 19	*KRT19*	rs11550883 rs4602 rs4601	Enhancer	p.Phe508del homozygous	↑PD severity		CGA	Gisler et al. [32]

Lymphotoxin alpha	*LTA*	rs909253 (+252A/G)	Enhancer	p.Phe508del homo and heterozygous	↑PD severity		CGA	Corvol et al. [24]

Mannose-binding lectin 2	*MBL2*	rs11003125	Enhancer	p.Phe508del homo and heterozygous	↑PD severity ↑Airway colonization with *P. aeruginosa* ↓Age (years) of the first isolation of *P. aeruginosa* ↓FEV1 annually as determined by spirometry		CGA	Yokoyama et al. [47]
		(XA/XA, YA/0, XA/0, 0/0)	Enhancer	p.Phe508del homo and heterozygous	↑PD severity		CC	Gravina et al. [50]
		Low-producing MBL2 genotypes	Suppressor	p.Phe508del homo and heterozygous	↑Age (years) of the first isolation of *P. aeruginosa*		CGA	McDougal et al. [39]

Macrophage migration inhibitory factor	*MIF*	rs5844572 (−794 CATT polymorphic repeats [MIF-CATT])	Suppressor	p.Phe508del homozygous	↓PD severity—Associated with a later onset of acute episodes, colonization by *P. aeruginosa*, and FEV1 score		CC	Melotti et al. [58]

Mucin 4, cell surface associated	*MUC4/MUC20*	rs3103933	Enhancer	p.Phe508del homo and heterozygous	↑PD severity		GWAS	Corvol et al. [17]
		rs2246771	Enhancer	p.Phe508del homo and heterozygous	↑PD severity		WGS	Zhou et al. [83]

Mucin 5 AC, oligomeric mucus/gel-forming	*MUC5AC*	VNTR	Enhancer	p.Phe508del homozygous	↑PD severity		CC	Guo et al. [52]

Protein phosphatase 2 phosphatase activator	*PPP2R4*	rs3118625	Suppressor	p.Phe508del homozygous	↓PD severity		CGA	Gisler et al. [32]

Protein phosphatase 2 scaffold subunit alpha	*PPP2R1A*	rs2162779	Suppressor	p.Phe508del homozygous	↓PD severity		CGA	Gisler et al. [32]

Sodium channel epithelial 1 subunit beta	*SCNN1B*	rs1391471777 (chromosome—16-p613L)	Suppressor	p.Phe508del homozygous	↓PD severity Positive impact on survival		WES	Agrawal et al. [77]

Sodium channel epithelial 1 subunit delta	*SCNN1D*	V541L, P579L	Suppressor	p.Phe508del homozygous	↓PD severity Positive impact on survival		WES	Agrawal et al. [77]

Solute carrier family 11 member 1	*SLC11A1*	rs17235416	Enhancer	p.Phe508del homo and heterozygous	↑PD severity ↑Airway colonization—Presence of *S. aureus*		CGA	Pereira et al. [70] and Pereira et al. [42]

Solute carrier family 26 member 9	*SLC26A9*	rs7512462	Enhancer	p.Phe508del homo and heterozygous	↑PD severity	↑Response to ivacaftor	OB	Strug et al. [76]

Solute carrier family 6 member 14	*SLC6A14*	rs3788766	Enhancer	p.Phe508del homo and heterozygous	↑Airway colonization—Presence of mucoid *P. aeruginosa* and *S. aureus*	↑Response to bronchodilator	CGA	Pereira et al. [70]
			Enhancer	p.Phe508del homo and heterozygous	↑Airway colonization—Presence of mucoid *P. aeruginosa*		CSS	Pereira et al. [70]
			Enhancer	p.Phe508del homo and heterozygous	↑PD severity		CGA	Mercier et al. [40]
		rs5952223	Enhancer	p.Phe508del homo and heterozygous	↑PD severity		GWAS	Corvol et al. [17]
		rs12839137	Suppressor	p.Phe508del homo and heterozygous	↓PD severity	↑Response to lumacaftor/ivacaftor	GWAS	Mésinèle et al. [7]

Solute carrier family 9 member A3	*SLC9A3*	rs17563161	Enhancer	p.Phe508del homo and heterozygous	↑Airway colonization—Earlier acquisition of *P. aeruginosa* ↑PD severity		CGA	Li et al. [37]
		rs4957061	Enhancer	p.Phe508del homo and heterozygous	↑Airway colonization—Earlier acquisition of *P. aeruginosa* ↑PD severity ↑Overall decline and mortality		CGA	Dorfman et al. [30]
		rs57221529	Enhancer	p.Phe508del homo and heterozygous	↑PD severity		GWAS	Corvol et al. [17]
	*SLC9A3/CEP72*	rs56108664	Enhancer	p.Phe508del homo and heterozygous	↑PD severity		WGS	Zhou et al. [83]

Synaptosome-associated protein 23	*SNAP23*	rs9302112	Suppressor	p.Phe508del homozygous	↓PD severity		CGA	Gisler et al. [32]

Signal transducer and activator of transcription 3	*STAT3*	Intronic microsatellite STAT3Sat	Suppressor	p.Phe508del homozygous	↓PD severity		CGA	Labenski et al. [36]

Syntaxin 1A	*STX1A*	rs4363087	Enhancer	p.Phe508del homozygous	↑PD severity		CGA	von Kanel et al. [46]

Taste 2 receptor member 38	*T2R38*	AVI allele	Enhancer	Not described	↑PD severity, including chronic sinonasal disease ↑Airway colonization—Presence of mucoid *P. aeruginosa*		CC	Castaldo et al. [49]

Transmembrane channel like 6	*TMC6*	rs34712518	Enhancer	p.Phe508del homo and heterozygous	↑Earlier acquisition of *P. aeruginosa* infection		WES	Emond et al. [79]

Toll-like receptor 1	*TLR1*	rs5743551	Enhancer	p.Phe508del homo and heterozygous	↑PD severity		CGA	Haerynck et al. [33]

Toll-like receptor 2	*TLR2*	rs1898830 rs5743708 rs3804100	Enhancer	p.Phe508del homo and heterozygous	↑PD severity		CGA	Haerynck et al. [33]

Toll-like receptor 5	*TLR5*	rs5744174	Enhancer	p.Phe508del homo and heterozygous	↑PD severity		CGA	Haerynck et al. [33]
		rs5744168	Suppressor	p.Phe508del homo and heterozygous	↓PD severity		CC	Blohmke et al. [48]

Transforming growth factor beta 1	*TGBβ1*	rs1800469	Suppressor	p.Phe508del homozygous	Reported better LFTs and increased survival in ESLD			Laubach et al. [75]

Tumor necrosis factor	*TNF-α*	308A>G	Enhancer	p.Phe508del homo and heterozygous	↑PD severity ↑Frequency of asthma ↑Levels of neutrophil elastase ↑Airway colonization—*P. aeruginosa*		CGA CC	Yokoyama et al. [47]
								Khorrami et al. [57]
		308G/A	No effect	p.Phe508del homo and heterozygous	No effect on airway colonization—*P. aeruginosa*		CGA	Corvol et al. [24]
		1031T/C	Enhancer	p.Phe508del homo and heterozygous	↑PD severity		CC	Khorrami et al. [57]

Tumor necrosis factor receptor	*TNFR1*	rs767455	Enhancer	p.Phe508del homozygous	↑PD severity, ↑FEV(1) decline		CC	Hassanzad et al. [53, 54]
	*TNFR2*	587T/G, 857 C/T	Enhancer	p.Phe508del homozygous	↑PD severity, ↑FEV(1) decline		CC	Hassanzad et al. [53, 54]

*Note:* The upward arrow symbol indicates an increase. The downward arrow symbol indicates a decrease.

Abbreviations: BMI, body mass index; CC, case control; CGA, candidate gene study; COPD, chronic obstructive pulmonary disease; CSS, cross-sectional study; dbSNP, the single nucleotide polymorphism database; ESLD, end-stage liver disease; FEV, forced expiratory volume; GWAS, genome-wide association study; LFTs, lung function tests; OS, observational study; PD, pulmonary disease; rs ID, reference identifier submitted to the SNP database; WES, whole exome sequencing.

**Table 2:** Summary of investigated gene modifiers associated with the gastroenterological system and their characteristics, including relevant references.

**Name of gene**	**Gene abbreviation**	**Polymorphism (if described—Indicated as dbSNP rs ID, the base/amino position and nucleotide/amino acid change, or variant type)**	**Enhancer or suppressor GM**	***CFTR* genotype**	**Clinical impact**	**Studies conducted**	**References**
Adrenoceptor beta 2	*ADRB2*	Arg16Gly	Enhancer	p.Phe508del homozygous	↑PI	CSS	Marson et al. [68, 69]

ATPase H+/K+ transporting nongastric alpha2 subunit	*ATP12A*	rs61948108	Enhancer	p.Phe508del homo and heterozygous	↑MI	GWAS	Gong et al. [18]

Calcium sensing receptor	*CASR*	V149I and N189D	Enhancer	p.Phe508del homo and heterozygous	↑Risk of recurrent and CP	CC	Sofia et al. [63]

CDK5 regulatory subunit associated protein 1 like 1	*CDKAL1*	rs7754840, rs7756992	Enhancer	p.Phe508del homo and heterozygous	↑Risk of CFRD	GWAS	Blackman et al. [16]

Cyclin-dependent kinase inhibitor 2A	*CDKN2A/B*	rs1412829	Enhancer	p.Phe508del homo and heterozygous	↑Risk of CFRD	GWAS	Blackman et al. [16]

Chymotrypsin C	*CTRC*	K172E	Enhancer	p.Phe508del homo and heterozygous	↑Risk of recurrent and CP	CC	Sofia et al. [63]

Cytochrome P450 family 11 subfamily B member 2	*CYP11B2*	rs6981918	Enhancer	p.Phe508del homo and heterozygous	↑Risk of CFRD	GWAS	Blackman et al. [16]

Homeostatic iron regulator	*HFE*	C282Y	Enhancer	p.Phe508del homo and heterozygous	↑CFRD, ↑MI ↑DIOS	CGA	Smith et al. [44]
		H63D	Enhancer	p.Phe508del homo and heterozygous	↑DIOS	CGA	Smith et al. [44]

Insulin-like growth factor 2 MRNA binding protein 2	*IGF2BP2*	rs1470579, rs4402960	Enhancer	p.Phe508del homo and heterozygous	↑Risk of CFRD	GWAS	Blackman et al. [16]

Keratin 18 pseudogene 33	*KRT18P33*	rs11902125	Enhancer	p.Phe508del homo and heterozygous	↑Risk of CFRD	GWAS	Blackman et al. [16]

Latrophilin-3	*LPHN3*	rs995447	Enhancer	p.Phe508del homo and heterozygous	↑Risk of CFRD	GWAS	Blackman et al. [16]

Methionine sulfoxide reductase A	*MSRA*		Enhancer	p.Phe508del homo and heterozygous	↑MI	CGA	Henderson et al. [35]

NCK-associated protein 1 like	*NCKAP1L*	rs4759088	Enhancer	p.Phe508del homo and heterozygous	↑Risk of CFRD	GWAS	Blackman et al. [16]

Serpin family A member 1	*SERPINA1*	rs28929474, rs17580	Enhancer	p.Phe508del homo and heterozygous	↓Age at CFLD onset ↑ESLD, ↑Cirrhosis ↑Portal hypertension ↑Esophageal varices	CGA	Boëlle et al. [22, 23]

Solute carrier family 9 member A3	*SLC9A3*	rs17563161	Enhancer	p.Phe508del homo and heterozygous	↑GI complications—Lowest age for onset of digestive symptoms, including MI	CGA	Li et al. [37]
				p.Phe508del homo and heterozygous	↑GI complications—Lowest age for onset of digestive symptoms, including MI, DM, and PI	CSS	Pereira et al. [70]
		rs6864158	Enhancer	p.Phe508del homo and heterozygous	↑Susceptibility to MI	GWAS	Sun et al. [19]

Solute carrier family 6 member 14	*SLC6A14*	rs3788766	Enhancer	p.Phe508del homo and heterozygous	↑GI complications—Lowest age for onset of digestive symptoms, including MI	CGA	Li et al. [37]
			Enhancer	p.Phe508del homo and heterozygous	↑MI	GWAS	Gong et al. [18]
			Enhancer	p.Phe508del homo and heterozygous	↑Susceptibility to MI	GWAS	[19])

Solute carrier family 26 member 9	*SLC26A9*	rs4077468	Enhancer	p.Phe508del homo and heterozygous	↑Susceptibility to MI	GWAS	Sun et al. [19]
			Enhancer	p.Phe508del homo and heterozygous	↑Risk of CFRD	GWAS	Blackman et al. [16]
			Enhancer	p.Phe508del homo and heterozygous	↑Risk of CFRD	GWAS	Aksit et al. [15]
		rs7512462	Enhancer	p.Phe508del homo and heterozygous	↑GI complications—Lowest age for onset of digestive symptoms, including MI and pancreatic damage	CGA	Li et al. [37]
			Suppressor	p.Phe508del homozygous	↑Age of diabetes onset	GWAS	Lam et al. [84]
			Enhancer	p.Phe508del homo and heterozygous	↑Risk of CFRD	GWAS	Blackman et al. [16]
		rs7549173	Enhancer	p.Phe508del homo and heterozygous	↑MI	GWAS	Gong et al. [18]
		rs4077469, rs7415921, rs1874361, rs7555534, rs7419153	Enhancer	p.Phe508del homo and heterozygous	↑Risk of CFRD	GWAS	Blackman et al. [16]

Transcription factor 7 like 2	*TCF7L2*	rs34872471	Enhancer	p.Phe508del homo and heterozygous	↑Risk of CFRD	GWAS	Blackman et al. [16]
		rs7903146	Enhancer	p.Phe508del homo and heterozygous	↑Risk of CFRD	GWAS	Aksit et al. [15]

Tumor necrosis factor	*TNF-α*	308GG, 3089GA	Enhancer	p.Phe508del homo and heterozygous	↑LD development ↑Cirrhosis development	CC	Shmarina et al. [62]
		1031T/C	Enhancer	p.Phe508del homo and heterozygous	↑GI complications including MI, rectal prolapse, stomach pain, fat in the stools, and greasy stools	CC	Khorrami et al. [57]
	*TNF-β* (LT-*α*)	252AA, 252AG	Enhancer	p.Phe508del homo and heterozygous	↑LD development ↑Cirrhosis development	CC	Shmarina et al. [62]

Chitinase 3 like 1	*CHI3L1*	rs4950928	Enhancer	p.Phe508del homo and heterozygous	↑Dysglycaemia	OS	Coriati et al. [72]

Glutathione S-transferase theta 1	*GSTT1*	Deletion	Enhancer	p.Phe508del homo and heterozygous	↑GI complications including DI, PI, and MI	CSS	de Lima Marson et al. [64]

Serine protease 1	*PRSS1*	rs3757377	Enhancer	p.Phe508del homo and heterozygous	↑MI	GWAS	Gong et al. [18]
		rs192452846	Enhancer	p.Phe508del homo and heterozygous	↑Risk of recurrent and CP	CC	Sofia et al. [63]

Serine protease 2	*PRSS2*	T230I and K98X	Enhancer	p.Phe508del homo and heterozygous	↑Risk of recurrent and CP	CC	Sofia et al. [63]
	*PTMA*	rs838440, rs838455	Enhancer	p.Phe508del homo and heterozygous	↑Risk of CFRD	GWAS	Aksit et al. [15]

*Note:* The upward arrow symbol indicates an increase. The downward arrow symbol indicates a decrease.

Abbreviations: BMI, body mass index; CC, case control; CFRD, cystic fibrosis–related diabetes; CGA, candidate gene study; CP, chronic pancreatitis; CSS, cross-sectional study; DB, diabetes mellitus; dbSNP, the single nucleotide polymorphism database; GI, gastrointestinal; GWAS, genome-wide association study; LD, liver disease; MI, meconium ileus; OS, observational study; PI, pancreatic insufficiency; rs ID, reference identifier submitted to the SNP database; WGS, whole genome sequencing.

**Table 3:** Summary of investigated gene modifiers associated with the reproductive, musculoskeletal, and other systems and their characteristics, including relevant references.

**System**	**Name of gene**	**Gene abbreviation**	**Polymorphism (if described—Indicated as dbSNP rs ID, the base/amino position and nucleotide/amino acid change, or variant type)**	**Enhancer or suppressor GM**	***CFTR* genotype**	**Clinical impact**	**Studies conducted**	**References**
Reproductive	Endothelin receptor type A	*EDNRA*	rs5335	Enhancer	p.Phe508del homozygous	↑CBAVD penetrance	CGA	Havasi et al. [34]
	MicroRNA 126	Hsa-let-7e	rs52196480	Enhancer	p.Phe508del homozygous	Recurrent pregnancy loss	CGA	Endale Ahanda et al. [31]
	Transforming growth factor	*TGF*	rs1982073	No effect	p.Phe508del homozygous	Does not affect CBAVD penetrance	CGA	Havasi et al. [34] Havasi et al. [34]
			rs1800471	No effect	p.Phe508del homozygous	Does not affect CBAVD penetrance	CGA	

Musculoskeletal	Glutathione S-transferase Pi 1	*GSTP1*	313A>G	Enhancer	p.Phe508del homo & heterozygous	↑Risk of osteoporosis Indications of increased risk of cancer	CSS	de Lima Marson et al. [64]
	Tumor necrosis factor	*TNF-α*	863CA 308A>G polymorphism	Enhancer	p.Phe508del homo and heterozygous	↓Bone density	CC	Shmarina et al. [62]
			1031T/C	Enhancer	p.Phe508del homo and heterozygous	Associated with poor growth	CC	Khorrami et al. [57]
		*TNF-β* (LT-*α*)	252AA	Enhancer	p.Phe508del homo and heterozygous	↑Risk of osteoporosis	CC	Shmarina et al. [62]
	Solute carrier family 6 member 14	*SLC6A14*	rs3788766	Enhancer	p.Phe508del homo and heterozygous	↑Osteoporosis	CSS	Pereira et al. [70]
	Sphingosine-1-phosphate	*S1P*		Enhancer	p.Phe508del homozygous	↓Bone density	CC	Jourdain et al. [56]

Other		*CEACAM3*		Enhancer	Dizygous p.Phe508del-CFTR homozygous CF siblings	Overall disease severity ↑Risk of mortality	CC	Stanke et al. [45]
		*CEACAM6*	rs1549960–rs11548735	Enhancer	Dizygous p.Phe508del-CFTR homozygous CF siblings	Overall disease severity ↑Risk of mortality		
	Tensin 1	*TNS1*	rs3796028	Enhancer	p.Phe508del homozygous	↓BMI	WGS	Walton et al. [82]
			rs2571445	Enhancer	p.Phe508del homozygous	↓BMI	CC	Sanchez-Dominguez et al. [60]
			rs918949	Enhancer	p.Phe508del homozygous	↓BMI	CC	Blohmke et al. [48]
	Solute carrier family 6 member 14	*SLC6A14*	rs3788766^∗^TT	Enhancer	p.Phe508del homo and heterozygous	↓BMI	CSS	Pereira et al. [70]
	Mannose-binding lectin 2	*MBL*	MBL sufficient or (XA/XA, YA/XA, YA/YA)	Suppressor	p.Phe508del homozygous	↑BMI or better BMI percentiles A trend towards decreased mortality	OS	Laubach et al. [74]
			rs5030737	Enhancer	p.Phe508del homozygous	↑Risk of mortality measured by the median age of death ↓BMI	OS	Laubach et al. [74]
			rs1800450	Enhancer	p.Phe508del homozygous	↑Risk of mortality measured by the median age of death ↓BMI	OS	Laubach et al. [74]
			rs1800451	Enhancer	p.Phe508del homozygous	↑Risk of mortality measured by the median age of death ↓BMI	OS	Laubach et al. [74]

*Note:* The upward arrow symbol indicates an increase. The downward arrow symbol indicates a decrease.

Abbreviations: BMI, body mass index; CBAVD, congenital bilateral absence of the vas deferens; CC, case control; CGA, candidate gene study; CSS, cross-sectional study; GWAS, genome-wide association study; OS, observational study; WES, whole exome sequencing.

**Table 4:** Summary of investigated gene modifiers from 2010 onwards and corresponding previous replication study findings prior to 2010.

**System**	**Gene**	**Findings**	**Replication studies from 2010 onwards (conflicts or agrees with previous evidence)**	**References**
Pulmonary system	*ADRB2*	No significant relationship between Arg16Gly and Gln27Glu polymorphisms in ADRB2 and the response to bronchodilators	Conflicts	Hart et al. [85]
		CF patients with the Arg16Gly polymorphism had better spirometry, although Gln27Glu showed no effect	Conflicts	de Paiva et al. [86]
		↑PD severity and no association with albuterol	Agrees, however, conflicts with evidence surrounding bronchodilators	Büscher et al. [87]
		↑PD severity	Agrees	Steagall et al. [88]
	*DEFB1*	Chronic colonization	Conflicts	Tesse et al. [89]
	*GCLC*	↓PD severity	Conflicts	McKone et al. [90]
	*GSTO1-1*	No association was found for lung function	Conflicts	Drumm et al. [91]
		↑PD severity	Agrees	Hull and Thomson [92]
		No association	Conflicts	Feuillet-Fieux et al. [93]
		↑PD severity	Agrees	Flamant et al. [94]
	*GSTP1*	No association was found for lung function		Drumm et al. [91]
		No association		Feuillet-Fieux et al. [93]
		↑Lung severity		Flamant et al. [94]
		GSTP1-Ile105-encoding allele contributes to hepatic dysfunction in CF		Henrion-Caude et al. [95]
	*GSTT1*	No association was found for lung function	Conflicts	Drumm et al. [91]
		No association	Conflicts	Feuillet-Fieux et al. [93]
		↑PD severity	Agrees	Flamant et al. [94]
	*IL-10*	↑PD severity	Agrees	Corvol et al. [96]
		↑Airway colonization with *Aspergillus fumigatus*	Agrees	Brouard et al. [97]
		↑Airway colonization with *P. aeruginosa*	Agrees	Tesse et al. [89]
	*IL-1β*	↑PD severity—Particularly in females	Agrees	Levy et al. [98]
		↑PD severity	Agrees	Corvol et al. [96]
	*IL-8*	↑PD severity	Agrees	
			Agrees	Corvol et al. [96]
	*MBL*	Also studied gene–gene interactions with TNFB1 reporting an association of ↑PD severity	Conflicts	Dorfman et al. [99]
		No effect	Conflicts	Drumm et al. [91]
		No effect	Conflicts	Carlsson et al. [100]
		Lung function was higher in the MBL deficiency-determining genotypes (XA/YO+YO/YO) than in the other genotypes	Agrees	Olesen et al. [101]
		No effect	Conflicts	Carlsson et al. [100]
		↑PD severity Earlier age of onset infection of *P. aeruginosa*	Agrees	Trevisiol et al. [102]
		↑PD severity Lower oxygen levels	Conflicts and agrees	Davies et al. [103]
		↓PD severity	Agrees	Yarden et al. [104]
	*MIF*	↓PD severity—Five CATT at the −794 promoter = decreased incidence of *P. aeruginosa* colonization and milder lung function deficit	Agrees	Plant et al. [105]
	*SCNN1B*	↑PD severity when exposed to smoke	^∗^Study evaluated gene modifiers-environment	Stanke et al. [106]
	*SERPINA1*	S and Z (deficient alleles)—Earlier age of onset infection however—No association with FEV1	^∗^SERPINA was investigated in liver studies post-2010	Döring et al. [107]
		S and Z		Mahadeva et al. [108]
		S and Z		Meyer, Braun, and Roscher [109]
		S and Z		Frangolias et al. [110]
		1237G>A		Henry et al. [111]
		No effect −1237G >A, S, and Z		Drumm et al. [91]
	*TGBβ1*	↑PD severity	Conflicts	Drumm et al. [91]
		↑PD severity	Conflicts	Arkwright et al. [112]
		↑PD severity when also exposed to smoke	Conflicts	Collaco et al. [113]
		↑PD severity	Conflicts	Arkwright et al. [112]
		No association with lung disease severity	Conflicts	Brazova et al. [114]
		↓PD severity	Agrees	Bremer et al. [115]
		↑PD severity	Conflicts	Corvol et al. [96]
	*TLR5*	No association	Conflicts	Urquhart et al. [116]
	*TNFR1*	↑PD severity when also exposed to smoke	Agrees	Stanke et al. [106]
	*TNF-α*	↑PD severity	Agrees	Arkwright et al. [112]
		↑PD severity	Agrees	Buranawuti et al. [117]
		↑PD severity	Agrees	Corvol et al. [96]
		↑PD severity	Agrees	Drumm et al. [91]
		↑PD severity	Agrees	Schmitt-Grohé et al. [118]
		↑PD severity when also exposed to smoke	Agrees	Stanke et al. [106]
		↑PD severity	Agrees	Yarden et al. [104]

Gastroenterological system	*ACE*	↑PD severity—High-producer ACE genotype predicts patients with CF who have an increased chance of developing portal hypertension	^∗^ACE was investigated in pulmonary studies post-2010	Arkwright et al. [112]
	*SERPINA1*	SERPINA1 Z—Developing severe liver disease with portal hypertension	Agrees	Bartlett et al. [119]

*Note:* The upward arrow symbol indicates an increase. The downward arrow symbol indicates a decrease. The asterisk symbol indicates that a previous study prior to 2010 has been conducted on this gene.

## Data Availability

All supplementary data, code, or other materials that are related to these articles can be found publicly on an online version.

## Supporting Information

Supporting Information Additional supporting information can be found online in the Supporting Information section. File S1 reports in detail the data extracted from the included articles of this systematic review.
