# Supplementary material for: Impact of Gene Modifiers on Cystic Fibrosis Phenotypic Profiles: A Systematic Review
Source: Hum Mutat. 2024 Oct 16;2024:6165547. doi: 10.1155/2024/6165547 (PMC11919198; doi:10.1155/2024/6165547)
Supplement: Supporting Information — Additional supporting information can be found online in the Supporting Information section. File S1 reports in detail the data extracted from the included articles of this systematic review. [file 6165547.f1.zip › Appendix A.pdf]

**Specific objectives**	i.Synthesize the current evidence of genetic variations in GMs relative to CF and determine the potential impacts of GMs on disease progression, pharmacokinetics and phenotypic profiles associated with CF.<br>ii.Document the current research exploring variations in the CFTR gene, GMs and gene modulator therapies.<br>iii.Identify potential GMs of interest for future drug therapies.

Keywords Cystic Fibrosis, CFTR, Gene Expression, Genetic Variations, Severity, Modifier Genes,

Options	Search terms
	("cystic fibrosis" OR CFTR) AND ("single nucleotide polymorphism" OR "modifier gene*" OR "candidate gene*" OR "gene polymorphism" OR SNP OR mutation OR indel OR "genetic variation*" OR "genomic variation*") AND (phenotype OR pathophysiolog* OR pathology OR aetiology OR etiology OR "clinical symptom*" OR manifestation*) AND (sever* OR progression OR decline OR accelerat*)

Phenotype

**No. articles (PubMed/Scopus/WoS/ScienceDirect)**	**Duplicate removed (PubMed/Scopus/Wos/ ScienceDirect)**	**Total Left**
**Total = 1,855**<br>PubMed<br>Scopus<br>WoS<br>Science direct<br>Cinahl	**Total = 991**<br>PubMed<br>Scopus – 156<br>WoS<br>Science direct<br>Cinahl	**Total = 864**<br>PubMed<br>Scopus<br>WoS<br>Science direct<br>Cinahl
