# Supplementary material for: Impact of Gene Modifiers on Cystic Fibrosis Phenotypic Profiles: A Systematic Review
Source: Hum Mutat. 2024 Oct 16;2024:6165547. doi: 10.1155/2024/6165547 (PMC11919198; doi:10.1155/2024/6165547)
Supplement: Supporting Information — Additional supporting information can be found online in the Supporting Information section. File S1 reports in detail the data extracted from the included articles of this systematic review. [file 6165547.f1.zip › Supplementary file S1.pdf]

	Title	Study method	CF genotype
1	The Epithelial Sodium Channel Is a Modifier of the Long-Term Nonprogressive Phenotype Associated with F508del CFTR Mutations	whole-exome sequencing	homozygous F508de
2	Impact of polymorphism of Multidrug Resistance-associated Protein 1 (ABCC1) gene on the severity of cystic fibrosis	Gene Based Ass	homozygous for the F508del mutation
3	YKL-40 as a clinical biomarker in adult patients with CF: Implications of a CHI3L1 single nucleotide polymorphism in disease severity	observational stu	delF508 Homozygote - 97 (52.4%) Heterozygote - 73 (39.5%)
4	Genome-wide association and linkage identify modifier loci of lung disease severity in cystic fibrosis at 11p13 and 20q13.2	GWAS	Patients in the GMS and 60% of the patients in the CGS and TSS are F508del homozygotes (F508del/F508del), while the remainder has other severe exocrine pancreatic CFTR genotypes
5	Influence of SNPs in Genes that Modulate Lung Disease Severity in a Group of Mexican Patients with Cystic Fibrosis	Gene Based Association Study	
6	A polymorphism in the 5' UTR of the DEFB1 gene is associated with the lung phenotype in F508del homozygous Italian cystic fibrosis patients	Gene Based Association Study	

7	Cytokine gene polymorphisms and severity of CF lung disease	longitudinal and cross-sectional data analyses examining the relationship between SNPs (TNF-α, IL-8, IL-10 and IL-1β) and clinical outcome measurements over a 13 year period	Homozygote f508del - 51% Heterozygote - 31%
8	EDNRA variants associate with smooth muscle mRNA levels, cell proliferation rates, and cystic fibrosis pulmonary disease severity	Candidate Gene Study	del508
9	Cystic fibrosis modifier genes related to Pseudomonas aeruginosa infection	Gene Based Ass[illegible]	272 (58.7%) DF508/DF508 155 (33.4%) DF508/other 36 (7.9%) other/other
10	DCTN4 as a modifier of chronic Pseudomonas aeruginosa infection in cystic fibrosis	Exome sequencing retrospective study	dF508 homozygous, n=335 (48.1%)

11	Variation in cilia protein genes and progression of lung disease in cystic fibrosis	Observational study	308 p.Phe508del homozygotes and 10 p.Phe508 heterozygotes
12	Association of cystic fibrosis genetic modifiers with congenital bilateral absence of the vas deferens	Gene Based Association Study	
13	FAM13A is a modifier gene of cystic fibrosis lung phenotype regulating rhoa activity, actin cytoskeleton dynamics and epithelial-mesenchymal transition	Gene Based Ass(	F508del
14	Analysis of DEFB1 regulatory SNPs in cystic fibrosis patients from North-Eastern Italy	case-control	35.5% were homozygote for the F508del CFTR mutation, 48.3% carried the F508del CFTR in heterozygosis with another CFTR mutation, and the remaining 16.2% presented other mutations.
15	Prostaglandin-endoperoxide synthase genes COX1 and COX2 - novel modifiers of disease severity in cystic fibrosis patients	Gene Based Ass(	homozygous for F508del mutation

16	Mucin variable number tandem repeat polymorphisms and severity of cystic fibrosis lung disease: significant association with MUC5AC	Case control of MUC genes	Phe508del homozygote

17	Mutations in the HFE gene can be associated with increased lung disease severity in cystic fibrosis	Gene Based Ass[illegible]	Wild-type -<br>F508del Homozygote - 54<br>F508del Heterozygote - 39<br>Other - 7<br>Unknown - 2<br><br>H63D -<br>F508del Homozygote - 19<br>F508del Heterozygote - 13<br>Other - 3<br>Unknown - 2<br><br>C282Y -<br>F508del Homozygote - 13<br>F508del Heterozygote - 9<br>Other - 1<br>Unknown - 1
18	Identification of IFRD1 as a modifier gene for cystic fibrosis lung disease	case control study	The GMSG cohort consists of CF patients homozygous for ΔF508 CFTR<br>CF twins and siblings (N =1,118) and their parents from 619 families were recruited by the CFTSS - 21 dizygous and 49 monozygous (MZ) twin pairs were included.

19	IL8 gene as modifier of cystic fibrosis: unraveling the factors which influence clinical variability	Longitudinal cross-sectional study	Modal - F508del/F508del F508del/–
20	Variants in the interleukin 8 gene and the response to inhaled bronchodilators in cystic fibrosis	Longitudinal cross-sectional study	Modal - F508del/F508del F508del/–
21	Impact of a Gap Junction Protein Alpha 4 Variant on Clinical Disease Phenotype in F508del Homozygous Patients With Cystic Fibrosis	Observational	homozygous F508del
22	Osteoclastogenesis and sphingosine-1-phosphate secretion from human osteoclast precursor monocytes are modulated by the cystic fibrosis transmembrane conductance regulator	case-control study	F508del

23	The hsa-miR-125a/hsa-let-7e/hsa-miR-99b cluster is potentially implicated in Cystic Fibrosis pathogenesis	Gene Based Ass(	p.F508del
24	Transcriptomic profile of cystic fibrosis patients identifies type I interferon response and ribosomal stalk proteins as potential modifiers of disease severity	Gene Based Ass(	F508del homozygous
25	Mannose-binding lectin gene as a modifier of the cystic fibrosis phenotype in Argentinean pediatric patients	Case control of MBL gene	75 were homozygous for the severe mutation ΔF508,<br>27 were heterozygous for ΔF508 together with another severe mutation,<br>and four patients carried two severe non-ΔF508 mutations.
26	Unraveling the complex genetic model for cystic fibrosis: pleiotropic effects of modifier genes on early cystic fibrosis-related morbidities	Gene Based Association Study	

27	Association of clinical severity of cystic fibrosis with variants in the SLC gene family (SLC6A14, SLC26A9, SLC11A1 and SLC9A3)	Gene Based Ass	Unknown/unknown (n= 40) 21.5% V562I/unknown (n= 1) 0.5% G576A/R668C (n= 1) 0.5% p.Glu528G > A/TG11- 5T (n= 1) 0.5% R334W/R334W (n= 1) 0.5% F508del/unknown (n= 33) 17.7% G542X/unknown (n= 1) 0.5% G542X/P205S (n= 1) 0.5% G542X/R334W (n= 1) 0.5% 622-2A > G/711 + 1G > T (n= 1) 0.5% G542X/I618T (n= 1) 0.5% D614G/unknown (n= 1) 0.5% F508del/D1152H (n= 1) 0.5% F508del/R334W (n= 2) 1.1%
28	The ACE gene D/I polymorphism as a modulator of severity of cystic fibrosis<br>Association of the D/D genotype with early initiation of clinical manifestationsbacterium Burkholderia cepacia colonization	cross-sectional	The patients' CFTR genotypes were: 44 patients (24.44%) without identified mutation, 51 (28.33%) with one identified mutation (25% F508del/-, 2.78% G542X/-,
29	Impact of MIF gene promoter polymorphism on F508del cystic fibrosis patients	Case-control	homozygous for F508del CFTR gene mutation

30	Interaction among variants in the SLC gene family (SLC6A14, SLC26A9, SLC11A1, and SLC9A3) and CFTR mutations with clinical markers of cystic fibrosis	cross-sectional, c	[illegible]<br>V562I/− (n=1) 0.6%<br>G576A/R668C (n=1) 0.6%<br>p.Glu528G>A/TG11-5T (n=1) 0.6%<br>R334W/R334W (n=1) 0.6%<br>F508del/− (n=29) 17.7<br>G542X/− (n=1) 0.6%<br>G542X/P205S (n=1) 0.6%<br>G542X/R334W (n=1) 0.6%<br>622-2A>C/711+1G>T (n=1) 0.6%<br>G542X/I618T (n=1) 0.6%<br>D614G/− (n=1) 0.6%<br>F508del/D1152H (n=1) 0.6%<br>F508del/R334W (n=2) 1.2%<br>F508del/F508del (n=52) 31.7%
31	Gene Expression in Transformed Lymphocytes Reveals Variation in Endomembrane and HLA Pathways Modifying Cystic Fibrosis Pulmonary Phenotypes	transcriptome wide asso study TWAS	p.Phe508del CF-affected homozygous
32	SLC6A14 Impacts Cystic Fibrosis Lung Disease Severity via mTOR and Epithelial Repair Modulation	Gene Based Association Study	

33	Clinical and molecular characterization of the potential CF disease modifier syntaxin 1A	Gene Based Ass(	homozygous for CFTR p.Phe508del
34	TAS2R38 is a novel modifier gene in patients with cystic fibrosis	case control study	Not described
35	Airways glutathione S-transferase omega-1 and its A140D polymorphism are associated with severity of inflammation and respiratory dysfunction in cystic fibrosis	Case Control	F508 homozygous (n = 45)<br>F508 heterozygous (n = 20)<br>other CFTR mutations (n = 41)
36	Genetic variations in toll-like receptor pathway and lung function decline in Cystic Fibrosis patients	Gene Based Ass(	F508del homozygous 48/89 (54%)<br>F508del compound heterozygous 41/89 (46%)

37	TNF-alpha polymorphisms as a potential modifier gene in the cystic fibrosis	Gene Based Ass	F508del
38	Association of TNF-alpha Gene Variants With Clinical Manifestation of Cystic Fibrosis Patients of Iranian Azeri Turkish Ethnicity	Case-control	30 were homozygous for ΔF508 and 50 carried no known mutation
39	Cystic fibrosis gene modifier SLC26A9 modulates airway response to CFTR-directed therapeutics	Association observational study	Two Phe508del allele (n= 1,759) G551D allele (n= 70)

40	TNFα -857 C/T and TNFR2 +587 T/G polymorphisms are associated with cystic fibrosis in Iranian patients	Case-control	Homozygous for ΔF508 mutation.
41	Investigating the Effect of TNF alpha (-863) and TNF alpha (-308) genes Polymorphism on the Progression of Disease in Patients with Cystic Fibrosis	case-control study	
42	TNF gene polymorphisms in cystic fibrosis patients: contribution to the disease progression	case-control	The CFTR genotype in 138 CF patients was homozygous or heterozygous for F508del (ΔF508).

43	Tensin 1 (TNS1) is a modifier gene for low body mass index (BMI) in homozygous [F508del]CFTR patients	Whole genome s	homozygous [delF508]CFTR compound heterozygous [delF508]CFTR non-[delF508]CFTR
44	Polymorphisms in ADRB2 gene can modulate the response to bronchodilators and the severity of cystic fibrosis	Cross-sectional study	
45	Mannose-Binding Lectin (MBL) and Gap Junction Protein Alpha 4 (GJA4) Gene Heterogeneity in Relation to Severity of Clinical Disease in Cystic Fibrosis	observational	homozygous F508del

46	Transforming Growth Factor ß1 and Gap Junction Protein Alpha 4 Gene Heterogeneity in Relation to the Severity of Clinical Disease in Cystic Fibrosis	observational	Homozygous F508del
47	Genetic Modifiers of Cystic Fibrosis-Related Diabetes Have Extensive Overlap With Type 2 Diabetes and Related Traits	genome-wide association study	Combination
48	AGER -429T/C is associated with an increased lung disease severity in cystic fibrosis	Gene Based Association Study	Homozygous F508del
49	Genetic modifiers of cystic fibrosis-related diabetes	genome-wide association study	Combination
50	TLR5 as an anti-inflammatory target and modifier gene in cystic fibrosis	Case-control	Combination

51	SERPINA1 Z allele is associated with cystic fibrosis liver disease	Gene Based Association Study	Combination
52	Ancestral haplotype 8.1 and lung disease severity in European cystic fibrosis patients	Gene Based Association Study	Combination
53	Genome-wide association meta-analysis identifies five modifier loci of lung disease severity in cystic fibrosis	genome-wide association analysis	Combination
54	Modulatory effect of the SLC9A3 gene on susceptibility to infections and pulmonary function in children with cystic fibrosis	Candidate gene study	Combination

55	Exome Sequencing of Phenotypic Extremes Identifies CAV2 and TMC6 as Interacting Modifiers of Chronic Pseudomonas aeruginosa Infection in Cystic Fibrosis	Exome sequencing	Combination
56	Exome sequencing of extreme phenotypes identifies DCTN4 as a modifier of chronic Pseudomonas aeruginosa infection in cystic fibrosis	Exome sequencing	Combination
57	Identification of SNPs in the cystic fibrosis interactome influencing pulmonary progression in cystic fibrosis	Candidate Gene Study	Homozygous F508del
58	Genetic association and transcriptome integration identify contributing genes and tissues at cystic fibrosis modifier loci.	genome-wide association analysis	Combination
59	Variation in MSRA modifies risk of neonatal intestinal obstruction in cystic fibrosis	Gene Based Association Study	Combination
60	Initial interrogation, confirmation and fine mapping of modifying genes: STAT3, IL1B and IFNGR1 determine cystic fibrosis disease manifestation	Gene Based Association Study	Homozygous F508del
61	Increased expression of anion transporter SLC26A9 delays diabetes onset in cystic fibrosis	genome-wide study	Homozygous F508del
62	Use of a modeling framework to evaluate the effect of a modifier gene (MBL2) on variation in cystic fibrosis	genome-wide study	Combination
63	Factors Predisposing the Response to Lumacaftor/Ivacaftor in People with Cystic Fibrosis	Gene Based Association Study	Combination

64	Population-based study of cystic fibrosis disease severity and haemochromatosis gene mutations	Gene Based Association Study	Combination
65	The tumor necrosis factor α (-308 A/G) polymorphism is associated with cystic fibrosis in Mexican patients	Case-control	Combination
66	Trans-heterozygosity for mutations enhances the risk of recurrent/chronic pancreatitis in patients with Cystic Fibrosis	Case-control	Combination
67	Hierarchical fine mapping of the cystic fibrosis modifier locus on 19q13 identifies an association with two elements near the genes CEACAM3 and CEACAM6	Gene Based Association Study	dizygous F508del-CFTR homozygous CF siblings
68	Multiple apical plasma membrane constituents are associated with susceptibility to meconium ileus in individuals with cystic fibrosis	Gene Based Association Study	NA
69	Increased Expression of Plasma-Induced ABCC1 mRNA in Cystic Fibrosis	Case control	Combination

70	Genetic Modifiers of Cystic Fibrosis Lung Disease Severity: Whole-Genome Analysis of 7,840 Patients	WGS	Combination

Modifier/Candidate Gene	Negative or positive I	Organ/clinical impact	Drug if applic.
ENaC Mutations<br>SCNN1D - chromosome 1<br>V541L, p579L, V541L<br>SCNN1B - chromosome 16<br>p613L	Positive	Pulmonary disease - preserved<br>FEV1 for at least 20 years<br>Positive impact on survival and<br>lung function	
ABCC1 - 5′FR/G-260C<br>(NCBI reference:<br>010393.16:g.15983174CNG)	Negative	rare CC genotype were<br>chronically colonized by<br>Pseudomonas A around 6 years<br>earlier	
chitinase 3-like 1 gene<br>CHI3L1 - rs4950928 SNP	Negative	lower lung function<br>more colonized with<br>Pseudomonas aeruginosa<br>dysglycemia	
chr11p13 is in an intergenic<br>region 3' to APIP and EHF<br>SNP rs6092179 is within an<br>LD block containing 4 other<br>SNPs (rs6024437, rs8125625,<br>rs6024454 and rs6024460),	Negative	Pulmonary	
a1AT<br>a1ACT<br>IL10<br>TNFa<br>MBL2 - Significant<br>ADRB2<br>NOS3<br>GSTP1	Negative	Pulmonary	
	Negative		

Cytokines-<br>IL-8,<br>TNF-α,<br>IL-1β<br>IL-10	negative ONLY for IL-8, IL-10 and IL-1β,	Lung disease
EDNRA - rs5335	Negative	Pulmonary disease severity
Clinically relevant :<br>Heme oxygenase-1 (HMOX1) - rs2071749<br>complement factor 3 (C3) rs11569393<br><br>Investigated:<br>CFB - rs537160 & rs2072633<br>C3 - rs11569393, rs393770, & rs7257062<br>HMOX1 - rs2071746 & rs2071749<br>TLR4 - rs10759931	Negative	Pulmonary function and age at first infection with P. aeruginosa<br><br>heme oxygenase-1 (HMOX1) rs2071749 had the most significant effect on lung function - paeds<br>complement factor 3 (C3) rs11569393 and HMOX1 rs2071746 in the adult groups
DCTN4<br>rs11954652<br>rs35772018	Negative	worse long-term pulmonary disease and shorter survival, and chronic Pa infection

DNAH14 (rs3856154 and rs950210) and DNAAF3 (rs58824375)- Negative lung function<br>DNAH6 - preserved lung function (rs1192269 in DNAH6 and rs115366080 in DNAH14)	Negative & Positive	Pulmonary - obstructive lung disease
endothelin receptor type A polymorphism (EDNRA) (rs 5335, rs1801708)<br>transforming growth factor (TGF)-b1 (rs 1982073, rs 1800471)	Negative	congenital bilateral absence of the vas deferens (CBAVD penetrance)
FAM13A - rs7682431 SNP	Negative	Pulmonary<br>chronic obstructive pulmonary disease
DEFB1- three single nucleotide polymorphisms (SNPs) in the 5'-untranslated region of the DEFB1 gene (namely g 52G>A, g-44C>G and g-20G>A)	No effect	No significant differences were found for allele, genotype and haplo type frequencies of DEFB1 g-52G>A, g-44C>G and g 20G>A SNPs in CF patients stratified for Pseudomonas aeruginosa infection, as well as in patients with a severe and mild clinical phenotype or in patients stratified for CFTR genotypes
functional variants in prostaglandin-endoperoxide synthase 1 (COX1, PTGS1) and prostaglandin-endoperoxide synthase 2 (COX2, PTGS2)<br>Positive –765C and 8473C	Positive	Pulmonary

MUC1, MUC2 (rs10902076), MUC5AC, and MUC7	Negative	Pulmonary

		rapid decline in lung function and are at increased risk of diabetes mellitus and gastrointestinal complications
HFE -<br>C282Y mutation<br>H63D substitution	Negative	CFRD<br>MI<br>DIOS
IFRD1 - rs7817, rs3807213, rs6968084		Pulmonary - significantly associated with variation in neutrophil effector function

IL8 - (rs4073, rs2227306 and rs2227307)	Negative	Pulmonary but can also contribute to lupus nephritis, periodontitis, breast cancer, lung diseases, prostate diseases, ovarian diseases	
IL8 - (rs4073, rs2227306 and rs2227307)	Negative	the IL-8 gene variants (and possibly in other genes, which modulate the inflammatory lung response) may potentiate or minimize the effect of BD and also influence the response to the medication.	Inhaled bronchodilators
gap junction proteins (GJA) GJA1/GJA4	Positive and negative	GJA4 (In carriers of the A allele) variant is associated with significantly better protection against end-stage lung disease and superior pulmonary function test results in F508del homozygous patients. GJA4 variant (rs41266431), homozygous G variant carriers (n = 84/116; 72.4%) had poorer pulmonary function (FVC% pred: mean 78/86, $p < 0.040$) and survival to end-stage lung disease was lower	
sphingosine-1-phosphate (S1P)	Negative	Bone - osteoclastogenic capacity of peripheral blood-derived monocytes contribute to low bone mineral density observed in the CF population	

microRNAs (miRNAs) in hsa-miR-126<br>SNP1 - A C-to-T transition was found for nt52196076 in hsa-let-7e (rs376594280)<br>SNP2 - A G-to-A substitution for nt52196409 (rs41275794)<br>SNP3 - T-to-C polymorphism at position (nt)52196453 (dbSNP id: rs12976445)	Negative	increased PI3K/Akt pathway activity causing inflammation<br>SNP3 (rs12976445) is associated with lower levels of hsa-miR-125a and ERbB2 over-expression in breast cancer patients<br>SNP2 and SNP3 correlated with recurrent pregnancy loss
Overexpressed - EPB41L4B, LOC644172,C4BPA, and ZNF683	Positive	Increased expression of EGR1 was found to be significantly associated with mild lung disease
MBL2	Negative	MBL insufficiency was associated with a 3.5-fold risk of having a severe phenotype.<br>It was also associated with an earlier onset of infection with P. aeruginosa
SLC26A9 - rs7512462<br>SLC9A3 - rs17563161<br>SLC6A14 - rs3788766	Negative	SLC26A9 was pleiotropic for meconium ileus and pancreatic damage<br>SLC9A3 for meconium ileus and lung disease severity<br>SLC6A14 for meconium ileus and both lung disease and age at first P. aeruginosa infection

SLC6A14 - rs3788766<br>SLC26A9 - rs7512462<br>SLC11A1 - rs17235416<br>SLC9A3 - rs17563161	Negative	SLC6A14 - rs3788766 - rs3788766*TT with lowest body mass index, presence of mucoid P. aeruginosa, and S. aureus, highest Bhalla score, rs3788766*CT with early start of pulmonary symptom and osteoporosis<br>SLC26A9 - rs7512462 - rs7512462*TT with best response to the inhaled bronchodilator for FEV1, rs7512462*CC with highest Shwachman-Kulczycki score<br>SLC11A1 - rs17235416 - rs17234516*Normal allele with lowest value of SaO2 and S. aureus<br>SLC9A3 - rs17563161 - rs17563161*GG with lowest age for onset of digestive symptoms (OR = 2.564; 95%CI = 1.234–5.33).	rs3788766*CC best response with bronchodilator
ACE gene D/I polymorphism	Negative	early initiation of clinical manifestations - Burkholderia cepacia colonization<br>higher risk for chronic infection with BC and deterioration of lung function	
MIF -<br>MIF-CATT 5-5<br>MIF-CATT 5-6<br>MIF-CATT 5-7<br>MIF-CATT 6-6<br>MIF-CATT 6-7<br>MIF-CATT 7-7	Positive	slower rate of lung function decline	NA

SLC6A14 - rs3788766*<br>SLC26A9 - rs7512462*<br>SLC11A1 - rs17235416*<br>SLC9A3 - -rs17563161*	Negative	pancreatic insufficiency (PI)<br>presence of mucoid<br>Pseudomonas aeruginosa<br>Pulmonary
LPAR6, a G protein coupled receptor,	Negative	lung disease severity by<br>(1) endomembrane function<br>(2) HLA class I genes<br>(3) endoplasmic reticulum stress response genes
SLC6A14 - rs3788766 single nucleotide polymorphism (SNP) rs3788766, located within SLC6A14 promoter, minor allele G being deleterious	negative	lung disease

STX1A - rs4363087 (c.467-38A>G) - clinically relevant<br>rs11541454<br>rs2228607<br>rs35459363<br>rs45549734	Negative	Pulmonary function
T2R38	Negative	severity of chronic sinonasal disease<br>occurrence of chronic Pseudomonas aeruginosa colonization
GSTO1-1 - Glutathione S-transferase omega-1	Negative	Pulmonary<br>COPD<br>cancer cell survival or in the inflammatory response.
TLR1 rs5743551<br>TLR2 rs1898830, rs5743708, and rs3804100<br>TLR3<br>TLR4<br>TLR5 rs5744174<br>TLR6<br>TLR7<br>TLR8<br>TLR9<br>TLR10<br>CD14,<br>LBP	Negative	Lung function rapidly descreasing FEV1%

TNF-α -308A>G polymorphism	Negative	Pulmonary<br>The AA genotype for -308G>A polymorphism was a risk factor for early gastrointestinal symptoms (OR=5.98, 95%CI=1.06-49.68) and protection for the first Pseudomonas aeruginosa<br>first P. aeruginosa, GA genotype was a risk factor<br>-308G>A polymorphism alleles, the G allele was a risk factor for early pulmonary symptoms yet showed better transcutaneous oxygen saturation<br>The A allele was a protective factor for early pulmonary symptoms (OR=12.26, 95%CI=0.08-0.89) and P. aeruginosa (OR=12.15, 95%CI=0002-0007), however, the same allele was a risk factor for worst transcutaneous oxygen saturation	
TNF-α (-1031T/C and -308G/A)	Negative	Pulmonary<br>Gastrointestinal<br>Poor Growth	
SLC26A9 - rs7512462		Pulmonary	increased response to ivacaftor

TNFα −857 C/T rs1799724 and -238A/G, rs361525 TNFR1 (+36A/G, rs767455) TNFR2 (+587T/G, rs1061622)	Negative	Pulmonary function - lower FEV1
TNF-α-308 GA TNF α - 863CA	Negative	pulmonary disease
TNF-α–308A TNF-α–308GG – LT-α + 252AA & TNF-α–308GA – LT-α + 252AG	Negative	TNF-α–308A -increased frequency of asthma, higher levels of neutrophil elastase, decrease of bone density TNF-α–308GG – LT-α + 252AA & TNF-α–308GA – LT-α + 252AG were unfavorable with regard to liver disease development<br><br>low TNF production show a higher frequency of tuberculosis and virus hepatitis infection.

TNS1: rs3796028; rs2571445: and rs918949		low BMI	
ADRB2 - Arg16Gly & Glu27Gln polymorphisms	Negative	Arg16Gly polymorphism in ADRB2 gene was associated with pancreatic insufficiency lower age at the first isolation of the Pseudomonas aeruginosa	Bronchodilators
		Pulmonary function (FVC% pred, mean 78/85; $p < 0.055$) and survival to end-stage lung disease were lower ($p < 0.043$) for this variant (rs41266431) in carriers homozygous for the G variant	
Mannose-Binding Lectin (MBL)<br>Gap Junction Protein Alpha 4 (GJA4)	Negative for MBL ins	MBL-sufficient variants were associated with superior BMI and a trend for better survival than MBL insufficient variants.	

		SNP (rs41266431) in GJA4
Transforming Growth Factor ß1 and Gap Junction Protein Alpha 4	Posistive	For the TGBβ1 variant: 509 carriers of the C variant (CT + CC genotype, n = 105, 91.3%) had better LFTs (Forced expiratory flow at 75% of the FVC (FEF75% predicted: median 40/29.5, p < 0.015)
TCF7L2 - rs34872471<br>TCF7L2 - rs7903146<br>SLC26A9 - rs4077468<br>PTMA - rs838455<br>PTMA - rs838440	Negative	TCF7L2 - associated with Type 2 Diabetes<br>Increased risk of CF-related diabetes (CFRD)
AGER-429T/C	Negative	Increased lung disease severity<br>AGER -429 minor allele (C) was associated with poorer lung function by increased RAGE expression
SLC26A9 5' to the SLC26A9 - rs4077468, rs4077469, rs7415921, rs1874361, rs7512462, rs7555534, rs7419153<br><br>TCF7L2 (rs7901695, rs7903146)<br>CDKAL1 (rs7754840, rs7756992)<br>CDKN2A/B (rs1412829),and IGF2BP2 (rs1470579, rs4402960) were associated with an increased risk of CFRD<br>CYP11B2 - rs6981918<br>KRT18P33 - rs11902125<br>NCKAP1L - rs4759088<br>LPHN3 - rs995447	Negative	Cystic Fibrosis–Related Diabetes
TLR5 c.1174C>T single nucleotide polymorphism (rs5744168)	Positive	lung function and body weight improved health indicators in adults with CF.

SERPINA1 Z (rs28929474) - an increased risk of developing CFLD and related complications compared with noncarriers.<br>and S (rs17580)	Negative	age at CFLD onset and the development of CFLD-related complications (severe liver disease with cirrhosis, portal hypertension, esophageal varices)
LTA - 909253 - +252A/G<br>TNF - 1800629 -−308G/A	LTA - 909253 -lower lung function<br>TNF - 1800629 - Chronic P. aeruginosa colonization - no effect	lung disease severity<br><br>LTA - 909253 -lower lung function<br>TNF - 1800629 - Chronic P. aeruginosa colonization - no effect
MUC4/MUC20 rs3103933<br>SLC9A3 rs57221529<br>HLA Class II rs116003090<br>AGTR2/SLC6A14 rs5952223<br>EHF/APIP rs10742326	Negative	Lung disease - primary cause of mortality
SLC9A3Gene<br><br>SLC9A3gene (rs4957061) - associated with earlier acquisition of P seudomonas infection, substantially reduced lung function and accelerated rate of decline<br><br>rs2244223 rs3734141<br>rs9885312 rs3756711<br>rs12657631 rs6868535<br>rs925693<br>rs13153157 rs4957061<br>rs11955160 rs10475283<br>rs7726057 rs699644<br>rs4957066	Negative	Lung disease - primary cause of mortality

		Chronic Pseudomonas aeruginosa Infection<br>age-of-onset of chronic P. aeruginos	
CAV2 rs8940<br>TMC6 rs34712518	Negative	CAV2 with increased age-of-onset of P. aeruginosa airway infection<br>Variants in TMC6 with diminished age-of-onset of P. aeruginosa airway infection	
DCTN4 - rs35772018	Negative	DCTN4 missense variant was significantly associated with both early age of first P. aeruginosa positive culture and early age-at-onset of chronic P. aeruginosa	
PPP2R4 - rs3118625<br>SNAP23 - rs9302112<br>PPP2R1A - rs2162779<br>KRT19 - rs11550883 rs4602 rs4601	Negative	influencing pulmonary progression	
ATP12A - rs61948108<br>SLC26A9- rs7549173<br>SLC6A14 - rs3788766<br>PRSS1 - rs3757377	Negative	Meconium ileus	
MSRA	Negative	Meconium ileus	
STAT3,<br>IL1B - rs3917356 and rs4848306<br>and IFNGR1 - rs1327475 , rs9376269-rs1327475 and, rs1327475-rs9376268	Negative	pulmonary	
SLC26A9 - rs7512462	Positive	delays diabetes onset<br>age at onset of diabetes	
MBL2	Positive	Positive correlation of low producing MBL2 genotypes with earlier age of Pa infection	
SLC6A14 - rs12839137 - associated with the nutritional response.	Positive	change in lung function and nutritional status	Lumacaftor/ivacaftor

HFE mutations (C282Y and H63D) - CF patients possessing HFE mutations had significantly better iron stores<br>Patients with HFE mutations were more likely to have had childhood bowel obstruction<br>Diabetes was more common in HFE carriers	Negative	systemic iron stores, Pseudomonas aeruginosa infection, lung disease severity and prevalence of diabetes.
MBL2 exon 1 (A, B, C and D), NS<br>IL-8 promoter (2251 A/T) - NS<br>TNFa promoter (TNF1/TNF2) - Tumor Necrosis Factor a (-308 A/G)<br>SERPINA1 (PI*Z and PI*S)- NS	Negative - Tumor Necrosis Factor a (-308 A/G)<br>The rest - no effect	allele was significantly associated with CF patients
8 genes involved in the IPAT: PRSS1, PRSS2, SPINK1, CTRC, CASR, CFTR, CTSB and KRT8	Negative	risk of recurrent/chronic pancreatitis
CEACAM6 - rs1549960–rs11548735<br>CEACAM3	Negative	Disease severity
SLC6A14 - rs3788766<br>SLC26A9 - rs4077468<br>SLC9A3 - rs6864158	Negative	lung<br>susceptibility to meconium ileus
ABCC1 - rs504348	No effect	Pancreatic insufficient CF subjects showed increased colonization with any form of Pseudomonas aeruginosa (OR = 3.125, 95% CI: 1.192–8.190) and mucoid P. aeruginosa (OR = 5.075, 95% CI: 1.307–28.620) compared to the pancreatic sufficient group

AGTR2		
SLC6A14		
API		
EHFP		
CHP2		
PRKCB		
HLA		
MUC4		
MUC20		
SLC9A3		
CEP72	Negative	Pulmonary disease severity

Population/ country	Population/ country	Adults/children/both	Adults/children/both	Mean age if avail	Gender
Boston Children's Hospital.<br>North America	North America	Adult	Adult	52-67	Males
France<br>Belgium<br>Italy	Combo	6 to 40 years	Both	Paris 17.43±3.32<br>Brussels 21.33±9.65<br>Verona 24±9.44	Male/female<br>Paris 8/6<br>Brussels 29/22<br>Verona 60/78
Canada	Canada	26.8-38.4	adults	33.1	103 - males<br>85 - females
North America	North America	Both	Both		
Mexico	Mexico	Both	Both	Age at onset of clinical manifestations (years)<br><br>Mild Subgroup 2.2 +- 3.4<br>Severe Subgroup 1.2 +- 2.1	Mild Subgroup Female 45.0% (36/80)<br>Severe Subgroup 58.3% (35/60)
Italy	Italy		Did not report		

Australia	Australia	Children	Children	9.94 (9.5–10.5)	78 -male<br>80 - female
Combined study between Ireland, United Kingdom, Cleveland & Seattle	combination study		Both	Cleveland - 19.2<br>Seattle - 20.8<br>Ireland - 25.9	Cleveland - 54.8% male<br>Seattle - 52.1% male<br>Ireland - 51.2% male
Canada	Canada	Both	Both	Primary Cohort<br>Mean - 16.2<br>Range - 1.0 to 61.1<br><br>Secondary Cohort<br>Mean -<br>Paeds - 11.67 (Range - (6.02 to 18.50))<br>Adults - 26.9 (Range - (16.81 to 53.10))	Primary Cohort<br>Male - 259 (52.3%)<br>Female - 236 (47.7%)<br><br>Secondary Cohort<br>Male -<br>Paeds - 218<br>Adults - 172<br><br>Female -<br>Paeds - 201<br>Adults - 164
France	France	Children	Children	mean (yrs) 8.625<br>Range (2.375 - 17.87)	Males 339 (48.7

North America	North America	both with a range of 14–68 years	both	30.34 ± 0.57 years	191 males<br>190 females
Portugal<br>Spain<br>Turkey	Combo		Did not report		Male cohort
France	France	6.0–57.6	both	21	627 - males<br>595 - females
Italy	Italy		Did not report	NA	Cases<br>31 males<br>31 females
Poland	Poland	patients were classified into 4 age groups: 0–3 years (early childhood), 4–7 years (preschool), 8–13 years (pre-puberty), and >13 years (post-puberty).	both	age range: 2.0–33.	(42 female and 52 male)

North America	North America	MUC1<br>Severe cohort - 8 - 25<br>Mild cohort - 15 - 52<br>MUC2<br>Severe cohort - 8 - 25<br>Mild cohort - 15 - 52<br>MUC5AC<br>Severe cohort - 8 - 25<br>Mild cohort - 15 - 52<br>MUC7<br>Severe cohort - 8 - 25<br>Mild cohort - 15 - 54	both	MUC1<br>Severe cohort - 16.3 ± 4.1<br>Mild cohort - 28.1 ± 9.7<br>MUC2<br>Severe cohort - 16.4 ± 4.1<br>Mild cohort - 28.0 ± 9.7<br>MUC5AC<br>Severe cohort - 16.3 ± 4.2<br>Mild cohort - 28.0 ± 9.8<br>MUC7<br>Severe cohort - 16.5 ± 4.0<br>Mild cohort - 28.8 ± 9.8	MUC1<br>Severe cohort - Male; n(%) 110 (51%)<br>Mild cohort - Male; n(%) 159 (53%)<br>MUC2<br>Severe cohort - Male; n(%) 106 (49%)<br>Mild cohort - Male; n(%) 177 (54%)<br>MUC5AC<br>Severe cohort - Male; n(%) 100 (52%)<br>Mild cohort - Male; n(%) 149 (54%)<br>MUC7<br>Severe cohort - Male; n(%) 117 (50%)<br>Mild cohort - Male; n(%) 290 (55%)

Australia	Australia	Adults	adults	Wild-type - 28<br>H63D - 31<br>C282Y - 28	Wild-type - 63 males<br>H63D - 22 males<br>C282Y - 14 males
North America	North America	Both	Both	NA	NA

Brazil	Brazil		Both	Age in months : 1(	92 - Male<br>94 - female
Brazil	Brazil		both	Age in months : 1(	92 - Male<br>94 - female
Portugal<br>Amsterdam<br>Germany	Combo	Adults	adults	27 years	66 male<br>50 female
France	France	CF - 29 to 58<br>Control - 32 to 69	adults	CF - 42.6 (29 to 58)<br>Control - 53.2 (32 to 69)	NA

France	France	adults	adults	NA	NA
Germany	Germany	Adults	adults	Mild - 25<br>Severe - 21	Mild<br>10 - Male<br>6 - Female<br>Severe<br>8 - Male<br>8 - Female
Argentina	Argentina	1.7 to 19.8 years	both	6.5	44 - Male<br>62 - Female
Canada	Canada		Did not report		Females<br>Pediatric lung disease severity - 386<br>P. aeruginosa - 365<br>IRT - 64

Brazil	Brazil	Both	both	163; 15.87 ± 14.4[illegible]	male - 82 (48.5[illegible]
Brazil	Brazil		children		90 (50%) were male<br>90 (50%) were female
Italy	Italy	Both	both	Brussels - 21.5 (+/-) 9.4<br>Verona - 24.27 (+/-) 9.3	Brussels - Female - 22<br>Verona - Female - 78

Brazil	Brazil	Both	both	158; 15.96 ± 14.59 Range (0.25 to 77.67)	80 - Male 84 - Female
North America	North America	Both	Both	Severe 16.5 (+/-) 4.6 (Range 8-25) Mild 28.0 (+/-) 9.9 (Range 15-58)	Severe males 157 (49.5%) Mild males 221 (50.5%)
France	France		both		

Bernese	Switzerland	Both	both	age 6 to 20	
Italy	Italy	5–65 years	both	20 years	105 males<br>105 female
Italy	Italy		Did not report		
Belgium	Belgium	mix	Both	16 y 4 m<br>Range 5–40 y 9 m	Case -<br>(38 female/58 male)

Brazil	Brazil	one to 26 years	Both	9.71 years (± 6.06	24 - males<br>25 - females
Iran	Iran	Mar-24	both		of the 30 homozygous -<br>14 - Male<br>16 - Female
Canada and France	Combo	NA	Did not report	NA	NA

Iran	Iran		both	CF - 15<br>Control - 18	CF - 34 women and 36 men<br>Control - 38 women and 41 men
Iran	Iran	5-26 years	both	21±5.1 years old	
Russia	Russia	Both	both	mean age, 12.7 ± (	NA

				35.9 (19-74)	Male - 50%<br>Females - 50%
America	North America	Adults	Adults	homozygous [delF508]CFTR - 35.8 (19-61)<br>compound heterozygous [delF508]CFTR - 36.2 (19-74)<br>non-[delF508]CFTR - 35.1 (21-71)	homozygous [delF508]CFTR - Male 46.7%<br>compound heterozygous [delF508]CFTR- Male 54.8%<br>non-[delF508]CFTR- Male 45.5%
Brazil	Brazil	both	both	246.68 ± 168,73	Male - 60<br>Female - 62
Germany<br>The Netherlands	Combination	Both	Both	27.6 years	Male - 61<br>Female - 51

Germany<br>The Netherlands	Combination	Both	Both	27.2 years		65 male<br>and 50 female<br>Caucasian<br>subjects
Multiple registries	Both				5740	5740
France		Median age v	48% F	967 patients		
Many	Both			3,059 individuals		
Canada	Both			(n = 2219)		

France	Both	3328 CF patients	Pediatric (n = 597)<br>Adult (n =466)
France (n=230), Germany (n=95) and UK (n=79)	Both	404	
Multiple	Both	6,365 CF patients	
Canada	Children	1,004 pediatric patients.	

North America	Both			
North America	Both			
Switzerland	Both	53 male and 42 female		95 well-characterized p.Phe508del homozygous CF patients
International - Canada, the United States and France	Both		63 CF participants	
Canada & North America	Both			1,335 unrelated CF patients
European Cystic Fibrosis Twin and Sibling Stud	Both			762 individuals
North America	Both			762 individuals
North America	US CF Twin and Sibling Study.			788 patients
France	Both			765 pwCF

Australia	Both	86 individuals
Mexico	Both	81 CF patients
Italy	Both	48 patients affected by CF complicated by RP/CP
central Europe		
North America<br>France		3,763<br>participants
North America		23

North America
Canada
France

Total sample size	CF sample	Control sample	Measure of disease severity as an outcome
5	5	0	FEV1% (forced expiratory volume in 1 second) -for lung function
333	203	130 healthy control subjects	FEV1<br>Weight<br>Height<br>BMI<br>Age of cc by PA
188	188		FEV1
3,467 CF patient	Primary study - n=1,978<br>Replication - (n=557)		FEV
140 CF patients	141 CF patients<br><br>Mild Subgroup<br>Severe Subgroup	NA	a) the age (years) of the first isolation of Pseudomonas aeruginosa (P. aeruginosa)<br>b) the annual decline in forced expiratory volume during the first second (FEV1) as determined by spirometry<br>c) isolation of P. aeruginosa at admission<br>d) assessment using the Brasfield scoring system

158	158	nil	FEV1, BMI z-score, admitted days and NIH score
1,577			FEV1<br>FVC
Primary Cohort - 1674 individuals from 558 trios (558 patients and both parents)	Primary Cohort - 1674 individuals from 558 trios (558 patients and both parents)		
Secondary Cohort<br>Paeds (n = 419)<br>Adults (n = 336)	Secondary Cohort<br>Paeds (n = 419)<br>Adults (n = 336)	NA	FEV1<br>age at diagnosis<br>age at first infection with P. aeruginosa.
Replucation stuc	Primary group - 43<br>Primary replication analysis - 696	Primary group -	Age at first P. aeruginosa–positive culture<br>Age of onset of chronic P. aeruginosa<br>Age of onset of mucoid P. aeruginosa

381 persons			measure of lung function based on forced expiratory volume in 1 second (FEV1)
131	80	51	
1222	1222		Kulich normalized mortality adjusted CF-specific lung phenotype (KNoRMA)
192	62	130	Differences in FEV1<br>PA colonization<br>Frequency of PA colonization
94			microbiological tests of sputum or throat swabs, chest radiography evaluation using the Brasfield score, and forced expiratory volume in one second (FEV1%) as lung function measurement.<br>(DSS).

2287	MUC1<br>Severe cohort - 215<br>Mild cohort - 300<br>MUC2<br>Severe cohort - 216<br>Mild cohort - 327<br>MUC5AC<br>Severe cohort - 192<br>Mild cohort - 276<br>MUC7<br>Severe cohort - 234<br>Mild cohort - 527	FEV1* (% predicted) mean ± SD *<br>At time of enrollment into study

NA	163<br>Wild-type (n=102)<br>H63D (n=37)<br>C282Y (n=24)<br>Sixty-one (37.4%) subjects carried a HFE gene mutation (H63D: 33 heterozygotes, 4 homozygotes, C282Y: 20 heterozygotes, 1 homozygote and 3 H63D/ C282Y compound heterozygotes)	NA	CFTR genotype<br>age<br>best lung function (forced expiratory volume in 1 s [FEV1] and forced vital capacity [FVC]),<br>body mass index (BMI) in the previous 12 months,<br>CFRD,<br>CF-related liver disease (CFLD),<br>history of MI or DIOS<br>presence of Pseudomonas aeruginosa in routine sputum cultures.
1155	CF twins and siblings (N =1,118) and their parents from 619 families were recruited by the CFTSS -	37 participants -	Raw pulmonary function test data,<br>CFTR genotypes,<br>height<br>weight measurements<br>FEV1 was used to derive cross-sectional (MaxFEV1CF%)<br>longitudinal (AvgFEV1CF% and EstFEV1%Pred) measures,

186	186		transcutaneous arterial hemoglobin oxygen saturation (SaO2)<br>mucoid Pseudomonas aeruginosa<br>Pulmonary function markers (FEFMax, SaO2 and spirometric variables)<br>Shwachman–Kulczycki<br>Kanga<br>Balla
186	186		transcutaneous arterial hemoglobin oxygen saturation (SaO2)<br>mucoid Pseudomonas aeruginosa<br>Pulmonary function markers (FEFMax, SaO2 and spirometric variables)<br>Shwachman–Kulczycki<br>Kanga
116	116		over 3 years using pulmonary function tests,<br>body mass index,<br>Pseudomonas aeruginosa colonization,<br>diabetes mellitus,<br>survival to end-stage lung disease,<br>blood and sputum inflammatory markers
28	15	13	PBMCs were thawed, then seeded on glass cover slips<br>OCs resorption assay<br>S1P assays

94		94	*In-silico*
32		32	FEV1
106	106<br>(10 sibling pairs were included)	na	Shwachman score and lung function tests<br>Age at infection with P. aeruginosa and age at death were also recorded
	1661<br>Pediatric lung disease severity - 815<br>P. aeruginosa - 730<br>IRT - 126		Pediatric lung disease severity<br>P. aeruginosa<br>IRT

188		188	NA		(i) gender;<br>(ii) self-declared ethnicity (caucasoid and noncaucasoid);<br>(iii)severity scores such as:<br>(a) Shwachman-Kulczycki [evaluates the general state of the patient, through aspects of nutrition, general activity, physical exams and radiological findings.<br>(b) Kanga (assessment score of acute pulmonary exacerbation, which predicts the improvement or worsening of respiratory function and can evaluate the therapeutic effects.<br>(c) Bhalla (tomographic score to assist the assessment of pulmonary compromise, therapeutic effects and selection of patients for transplantation.<br>(iv) body mass index<br>(v) patient's age and age at diagnosis<br>(vi) first clinical pulmonary and digestive symptoms (months)<br>(vii) period until the first colonization by P. aeruginosa<br>(viii) isolated microorganisms
180					Kanga, Shwachman-Kulczycki and Bhalla<br>Nutritional status<br>BMI<br>Age of the diagnosis, the onset of pulmonary and digestive symptoms, and the first isolation of Pseudomonas aeruginosa, were used as markers of initiation of the disease.
323	189<br>Brussels - 51<br>Verona - 138			134	FEV1<br>cc by PA<br>Diabetes n (%) 11 (21.6%) 40 (29.0%) 0.36<br>BMI z score

	164		164	NA	(i) gender;<br>(ii) self-declared ethnicity (caucasoid and noncaucasoid);<br>(iii)severity scores such as:<br>(a) Shwachman-Kulczycki [evaluates the general state of the patient, through aspects of nutrition, general activity, physical exams and radiological findings.<br>(b) Kanga (assessment score of acute pulmonary exacerbation, which predicts the improvement or worsening of respiratory function and can evaluate the therapeutic effects.<br>(c) Bhalla (tomographic score to assist the assessment of pulmonary compromise, therapeutic effects and selection of patients for transplantation.<br>(iv) body mass index<br>(v) patient's age and age at diagnosis<br>(vi) first clinical pulmonary and digestive symptoms (months)<br>(vii) period until the first colonization by P. aeruginosa<br>(viii) isolated microorganisms
	754	754<br><br>Severe 317<br>Mild 437		NA	(KNoRMA) value<br>Age of Onset of Persistent Pseudomonas aeruginosa
large French CF cohort (n = 3,257)					Measurements of FEV1<br>To assess the lung disease severity, FEV1 were transformed to the Survival Adjusted Kulich Normalized (SaKnorm Z-value)

56 CF patients	56 CF patients	NA	lung clearance index (LCI),<br>forced expiratory volume in 1 s (FEV1),<br>forced expiratory flow (FEF) at 50% (FEF50)<br>specific airway resistance (sReff),<br>functional residual capacity and volume of trapped gas
305	210	95<br>(38 males; age: 7–66 years; median: 18 years)	sputum or oropharyngeal swab culture<br>modified Leeds criteria<br>Lund and Kennedy scale
150	106	44	we measured GSTO1-1 levels in sputum and ana- lyzed GSTO1-1 polymorphisms in blood samples
172	96	79 healthy contr	FEV1

49		49		age at onset of pulmonary and digestive symptoms<br>first isolation by P. aeruginosa<br>spirometry<br>Shwachman-Kulczycki & Kanga scores<br>transcutaneous oxygen saturation of hemoglobin
137		80	157	The diagnostic criteria included positive sweat tests and typical clinical findings of pulmonar and gastrointestinal disease
1829	Two Phe508del allele (n= 1,759)<br>G551D allele (n= 70)			forced expiratory volume in 1 second (FEV1) over a 3-year period.<br>For genetic association analysis a survival-adjusted averaged Cfspecific Kulich FEV1 percentile (calculated as a function of age, height and sex) that was normalized (SaKnorm)

	149	70	79	Chest radiographs were scored using Chrispin-Norman system<br>Schwachman score of disease severity was calculated<br>FEV1<br>FVC<br>Microbiological testing conducted by sputum or cough swabs
100 (41 patients and 47 members of control group remained until the end of the study),		50	50	O2 sat<br>FEV1<br>FVC<br>Schwachman index basis score<br>age at diagnosis time<br>starting point of the pulmonary disease
	228	198	130	Clinical, biological and functional data were obtained from hospital records from the previous 2 to 15 years.<br>Lung function was assessed by spirometry in children >4 years during periods of clinical stability.<br>Chronic airway colonization with Pseudomonas aeruginosa<br>Forced expiratory volume

98	98	NA	FEV<br>FVC<br>PI<br>Diabetes<br>BMI
122			Shwachman - Kulczycki, Kanga and Bhalla<br>forced expiratory volume in the first second[FEV1(%)]<br>forced expiratory flow<br>lower age at the first isolation of the Pseudomonas aeruginosa<br>spirometry<br>body mass index (BMI)<br>age at diagnosis
112	112		The clinical phenotype was assessed over three years using pulmonary function tests, body mass index, Pseudomonas aeruginosa colonization, diabetes mellitus, survival to end-stage lung disease, and inflammatory markers.

disease severity was assessed over 3 years using lung function tests (LFTs), body mass index, diabetes mellitus, colonization with Pseudomonas aeruginosa, survival to end-stage lung disease (ESLD), as well as distinct inflammatory biomarkers.

115 115

FEV1 was analyzed as CF-specific percentile adjusted on age, height and mortality.

35 patients with CF without pancreatitis and 80 unrelated healthy subjects

7840	KNoRMA phenotype
